# Circadian rhythm disruption in cardiovascular disease: a systematic review and meta-analysis of mechanistic evidence from animal models

**DOI:** 10.1186/s12916-025-04572-3

**Published:** 2026-01-09

**Authors:** Mrinal K. Das, Evi De Ryck, Ingrid L. Jorgensen, Shan Zienolddiny-Narui, Johanna Samulin Erdem

**Affiliations:** 1 National Institute of Occupational Health (STAMI), Pb 5330, Oslo, 0304 Norway; 2 Department of Public Health and Primary Care, KU Leuven, Louvain, Belgium

**Keywords:** Clock genes, Simulated shift work, Meta-analysis, Cardiac hypertrophy, Endothelial dysfunction, Animal models

## Abstract

**Background:**

Cardiovascular disease (CVD) remains the leading cause of death worldwide. While traditional risk factors are well-established, emerging evidence suggests shift work causing circadian rhythm disruption significantly contributes to CVD risk. This systematic review investigated molecular mechanisms linking circadian disruption with cardiovascular pathophysiology through in vivo models.

**Methods:**

We systematically searched Medline, Embase, and Web of Science through February 2025. Studies employing genetic (clock gene knockouts/mutations) or environmental (light phase shift, sleep deprivation) models of circadian disruption in vivo were included. Meta-analyses were performed for key cardiovascular indicators, and certainty of evidence was evaluated using a modified GRADE approach.

**Results:**

Among 9012 references, 34 studies met inclusion criteria. Following quality assessment for study design and reporting, 32 studies with low or moderate risk of bias were included in the synthesis. Meta-analyses revealed cardiac hypertrophy as the most robust finding, with high-certainty evidence for increased left ventricular mass-to-body weight ratio (LV/BW; SMD: 0.89, 95% CI: 0.38 to 1.39) and moderate-certainty evidence for increased cardiomyocyte size. These convergent organ and cellular-level findings, supported by elevated natriuretic peptides and pro-fibrotic markers, indicate circadian disruption contributes to pathological cardiac remodeling. Sensitivity analyses revealed low-certainty evidence for impaired systolic function, with significant reductions in ejection fraction (SMD: − 1.70, 95% CI: − 3.22 to − 0.17) and fractional shortening (SMD: − 1.60, 95% CI: − 2.71 to − 0.49). Low-certainty evidence was found for impaired endothelium-dependent vasorelaxation (SMD: − 2.72, 95% CI: − 4.90 to − 0.53) based on three genetic model studies with high heterogeneity and elevated triglyceride levels (SMD: 1.64, 95% CI: 0.07 to 3.21). Other markers showed very low-certainty evidence.

**Conclusions:**

This systematic review improves mechanistic understanding of CVD development following circadian misalignment by demonstrating cardiac hypertrophy as a major pathophysiological consequence in animal models. Cardiac structural changes at organ and cellular levels, supported by biomarkers of pathological remodeling, indicate circadian disruption contributes to adverse cardiac remodeling. Future animal research should prioritize standardized protocols, sex-balanced designs, and environmental models replicating human shiftwork patterns. Substantial epidemiological gaps remain, warranting further investigation in shift workers.

**Graphical Abstract:**

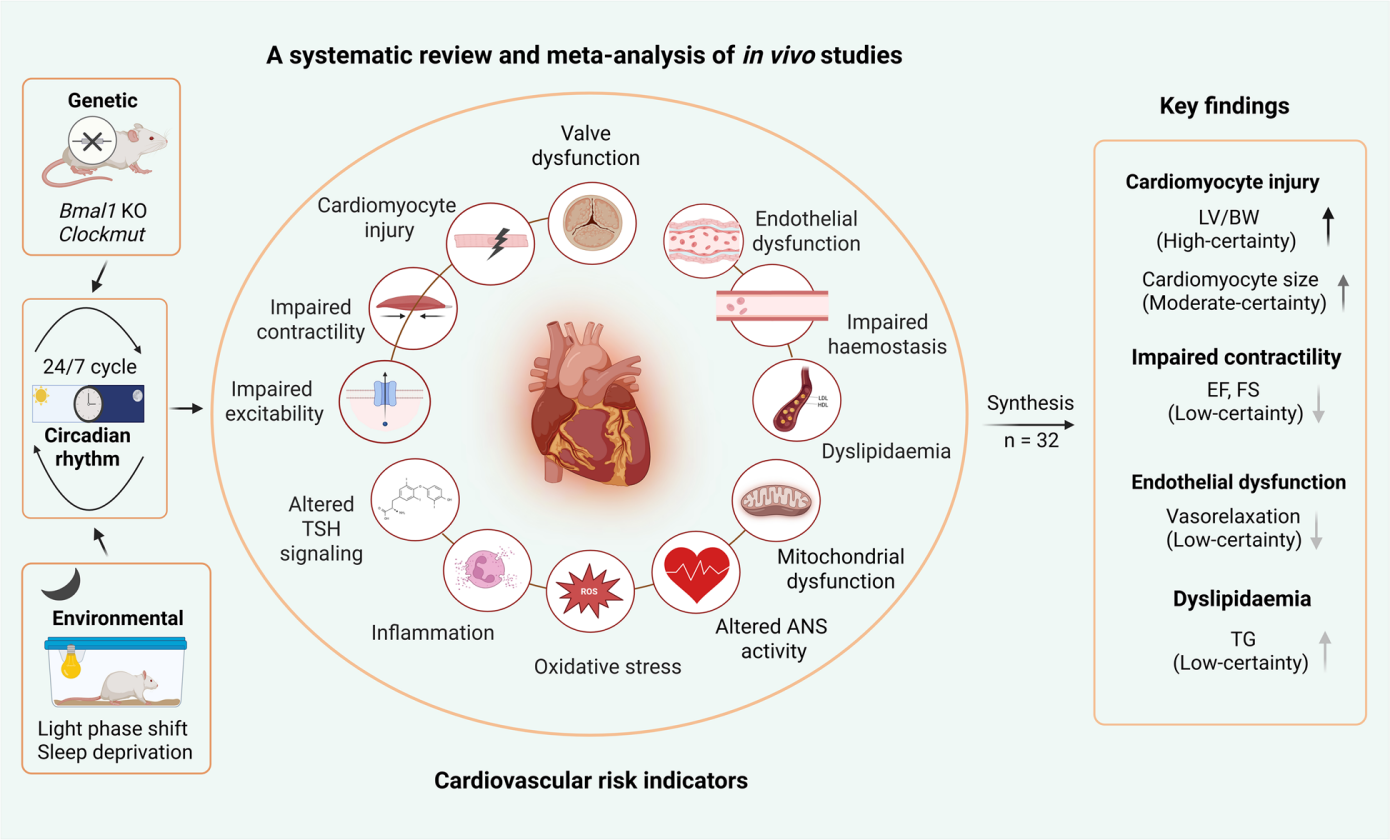

**Supplementary Information:**

The online version contains supplementary material available at 10.1186/s12916-025-04572-3.

## Background

Cardiovascular disease (CVD) is a multifaceted condition that remains the foremost cause of death worldwide, with approximately 17.9 million deaths annually [1]. While unhealthy lifestyles, such as poor dietary choices, sedentary behavior, and smoking, are the major CVD risk factors, emerging evidence suggests a relationship between shiftwork, particularly night shiftwork, and CVD risk. A recent meta-analysis of cohort studies by Su et al. reported that night shift work is associated with a 15% increased risk of cardiovascular mortality (risk ratio (RR): 1.18; 95% confidence interval (CI): 0.94–1.47, *I*^2^ = 81.3%) [2]. Another meta-analysis on shift workers substantiated this association, showing a 23% increased risk of myocardial infarction (RR: 1.23; 95% CI: 1.15–1.31, *I*^2^ = 0%) and a 24% increased risk of coronary events (RR: 1.24; 95% CI: 1.10–1.39, *I*^2^ = 85%) [3]. Manohar et al. also reported in their meta-analysis that shift workers presented a 31% greater risk of hypertension in cohort studies (RR: 1.31; 95% CI, 1.07–1.60, *I*^2^ = 90%) and a 10% higher risk in cross-sectional studies (RR: 1.10; 95% CI, 1.00–1.20, *I*^2^ = 85%) [4]. While the underlying mechanisms behind these associations have not been fully elucidated, disruption of circadian rhythms is considered a central contributor.

Circadian rhythms are 24-h biological cycles within organisms that regulate sleep-wake patterns and synchronize internal functions with external day-night cycles and environmental cues, such as light and feeding [5]. In mammals, circadian rhythms are controlled by a central clock, located in the suprachiasmatic nucleus (SCN) of the hypothalamus and peripheral clocks across various tissues. The SCN receives light cues through the retina and synchronizes peripheral clocks via neurohumoral signals, ensuring the coordination of physiological functions, including those in the heart and vasculature [6–8]. This regulation is driven by clock genes, such as circadian locomotor output cycles kaput (*Clock*), brain and muscle Arnt-like protein 1 (*Bmal1*), period (*Per1* and *Per2*), and cryptochrome (*Cry1* and *Cry2*), which form feedback loops that regulate the expression of genes involved in various biological processes. Thus, circadian rhythms are critical regulators of cardiovascular physiology, and peripheral clocks, which are present in each cardiovascular cell type [9–13], regulate various cardiovascular functions, such as endothelial function, blood pressure, heart rate, and vascular tone [14, 15]. While shift work, irregular sleep patterns, and misaligned light exposure are considered disruptors of circadian rhythms, the mechanisms by which these disruptions lead to cardiovascular diseases remain underexplored. Given the practical limitations of performing mechanistic studies in humans, animal models have become instrumental in exploring the biological and molecular impacts of circadian disruption in cardiovascular disease. In vivo studies in the literature have employed two main experimental approaches: genetic models and environmental models. Genetic models utilize knockout (KO) or mutation of clock genes including, but not limited to, *Bmal1* and *Clock*, with tissue-specific models providing precise targeting of clock components with minimal confounding effects [16–20]. Environmental models encompass light interventions such as light phase shifts that better recapitulate the intermittent circadian misalignment experienced by shift workers and constant light exposure that induces more severe disruption, as well as sleep disruption approaches including sleep deprivation and sleep fragmentation [21–24].

Despite the growing epidemiological evidence linking shift work to CVD, systematic evaluation of the mechanistic evidence from experimental models has not been comprehensively undertaken. While several narrative reviews have discussed circadian regulation of cardiovascular function [5, 25, 26], no systematic review has quantitatively synthesized the mechanistic evidence or formally assessed the certainty of this evidence using established frameworks. Previous systematic reviews in this area have focused primarily on epidemiological associations rather than biological mechanisms [3, 4, 27–29], leaving a critical knowledge gap in our understanding of how circadian disruption mechanistically contributes to cardiovascular pathophysiology. This knowledge gap limits the development of targeted interventions and biomarker strategies for shift workers at elevated cardiovascular risk.

This review aimed to systematically evaluate and quantitatively synthesize mechanistic evidence linking circadian disruption to CVD, focusing on key events including cardiac dysfunction, loss of vascular integrity, dyslipidemia, and inflammation. By including both genetic and environmental models of circadian disruption, this review seeks to provide a comprehensive overview of the current evidence and to advance our mechanistic understanding of circadian disruption as an independent risk factor for CVD.

## Methods

### Search strategy and eligibility criteria

A systematic literature search, based on an adapted PICO/PECO (Population, Intervention/Exposure, Comparator, Outcome) framework and according to the PRISMA (Preferred Reporting Items for Systematic Reviews and Meta-Analyses) 2020 guidelines [30], was performed on the molecular mechanisms and biomarkers related to CVD development and circadian disruption. The study is registered in PROSPERO: CRD42022337285. The PRISMA checklist is included in Additional file 1: Supplementary Table 1. The search was performed according to the search strategy outlined in Additional file 1: Supplementary Tables 2–4, using the Medline, Embase, and Web of Science databases through February 2025 in four intervals: 9 June 2022, 16 June 2023, 3 May 2024, and 20 February 2025, to ensure inclusion of the most recent evidence. The search strategy was intentionally broad to capture both epidemiological and animal studies. Retrieved articles were screened according to predefined PICO/PECO criteria and allocated into two separate reviews: a recently published meta-analysis on indicators of cardiovascular risk in night shift workers [27] and the present review focusing on mechanistic findings from animal models. Only studies directly meeting the PECO-defined inclusion criteria were retained. The PECO was defined as follows: “Population (P)” adult mammalian models including rodents, pigs, dogs, rabbits, and apes (disease models were excluded to minimize confounding effects from underlying pathologies); “Exposure (E)” circadian disruption induced through genetic modifications of clock genes (e.g., *Bmal1*, *Clock*, *Nr1d1/Nr1d2*, *Per*, *Cry* KO or mutations) or environmental interventions (light phase shifts, constant light exposure, sleep deprivation, sleep fragmentation); “Comparator (C)” wild-type littermate controls for genetic models or animals maintained under standard 12:12 h light–dark cycles for environmental models; and “Outcome (O)” cardiovascular risk indicators including cardiac functional parameters (ejection fraction, fractional shortening), structural markers (left ventricular mass, cardiomyocyte size, pathological hypertrophy), vascular function (endothelium-dependent vasorelaxation), and cardiovascular biomarkers (natriuretic peptides, lipid profiles, inflammatory markers). Outcome was defined according to the framework proposed by Lind et al. [31]. Detailed eligibility criteria for inclusion and exclusion are provided in Additional file 1: Supplementary Table 5. The search strategy was developed in collaboration with an experienced librarian (ILJ) who provided expertise in database-specific vocabularies, search term optimization, and validation of search comprehensiveness through iterative refinement and pilot testing.

Two independent reviewers manually screened all records using Covidence systematic review software (Veritas Health Innovation, Melbourne, Australia, www.covidence.org) to manage and organize the workflow. Duplicates were removed by Covidence, or manually by the reviewers. Inclusion and exclusion criteria were predefined according to the PECO framework in consultation with epidemiologists and cardiovascular specialists. Inclusion criteria: original studies; English language; in vivo studies on adult mammalian models including rodents, pigs, dogs, rabbits, and apes, where rodent models were considered mature at > 8 weeks of age (Population); light intervention, sleep deprivation, or modification of circadian genes (Exposure); control group defined as wild-type littermates or animals maintained under standard light conditions (Comparator); mechanistic evidence and cardiovascular biomarkers based on Lind et al. [31] and expert judgment, emphasizing potential novel biomarkers (Outcome). To ensure objectivity and consistency, screening was conducted independently by two reviewers. Disagreements were resolved through discussion until consensus was reached. If an agreement was not reached, a third reviewer was consulted. All reviewers had expertise in molecular and cellular cardiovascular biology, circadian research, and in vivo models.

Inter-rater reliability was assessed using Cohen’s kappa coefficient [32] for each of the four search iterations conducted between 2022 and 2025. Overall inter-rater agreement was calculated as the weighted average of kappa values across all search iterations, with weights proportional to the number of records screened in each iteration [33]. Agreement between the two independent reviewers was moderate for title/abstract screening (*κ* = 0.46, 95% CI 0.43–0.49; 84% agreement across 7395 records) and moderate for full-text screening (*κ* = 0.58, 95% CI 0.51–0.65; 79% agreement across 1148 records). Stable inter-rater agreement across all four iterations (no temporal trend) suggests consistent application of inclusion criteria by both reviewers throughout the 3-year review period.

**Fig. 1:**
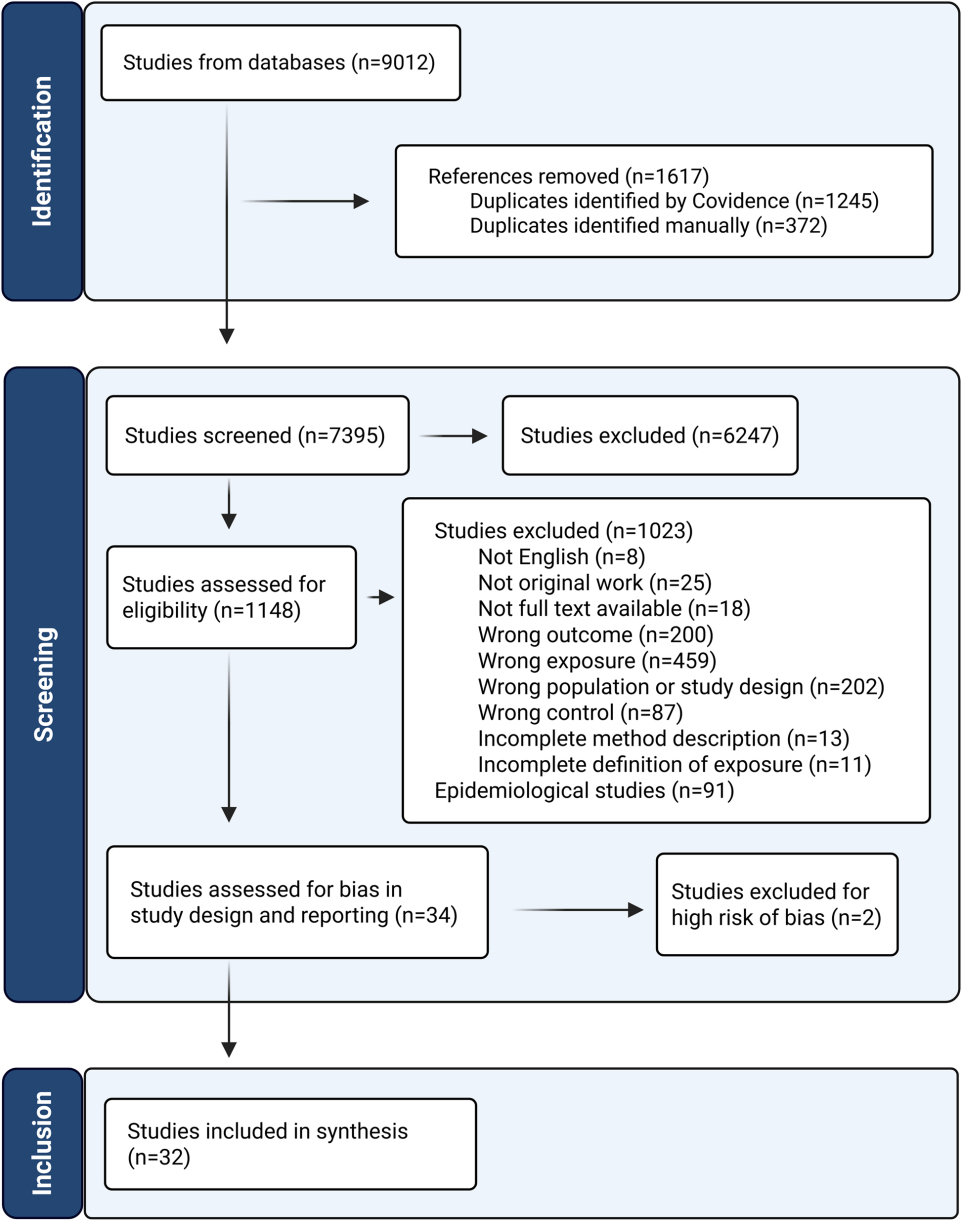
Prisma flowchart

Additionally, for the full-text screening, studies lacking full text or having incomplete methodology or statistics and studies on disease models were excluded. An overview of the screening and selection process, including exclusion reasons, is provided in the PRISMA flow diagram (Fig. 1). Studies that did not meet the predefined PECO criteria on Population or Exposure (wrong population or study design), Comparator (wrong control), and Outcome (wrong outcome) were excluded.

### Risk of bias assessment, data collection, and synthesis

Two experts assessed the risk of bias across four bias domains: (1) population; (2) intervention; (3) analytical; (4) design and reporting. Each bias domain was scored as low (L = 1), moderate (M = 2), or high (H = 3). Population bias was stratified by sample size (high: *n* ≤ 5, moderate: 6–10, low: > 10 per group). Small group sizes (≤ 5 animals) typically appear underpowered and more prone to random error and sampling variability [34, 35]. Studies with 6–10 animals per group represent common preclinical practice and provide improved reliability, while larger groups further reduce variance and outlier influence, although most preclinical studies remain underpowered by clinical standards [36]. Intervention bias was categorized according to the specificity and controllability of the circadian disruption. Tissue-specific genetic models and controlled light phase-shift paradigms were considered low risk due to their targeted manipulation with minimal systemic confounding. Global genetic models and sleep deprivation protocols were considered moderate risk due to broader physiological effects, while constant light and sleep fragmentation models were rated high risk because they engage multiple non-specific pathways including stress and metabolic responses [37]. Analytical bias was scored based on measurement reliability. Physical cardiac measures (e.g., EF, FS) were classified as low risk due to direct quantification with high reproducibility [38]. Protein biomarkers were rated moderate risk due to indirect measurement requiring sample processing and biological variability in expression levels (e.g., western blot, ELISA). mRNA-based markers (e.g., qPCR) were classified as high risk because transcript abundance shows only modest correlation with protein levels and may not reflect functional cardiovascular outcomes [39]. For assessment of study design and reporting bias, a modified ToxRTool [40] was used, in which non-applicable questions related to test substances were excluded (Additional file 2). According to the ToxRTool guidelines, the study bias was classified as category 1 (low), category 2 (moderate), or category 3 (high) and only studies with low or moderate study bias (ToxRTool categories 1 and 2) were included for data extraction. For quality assessment using the modified ToxRTool, inter-rater agreement was very strong (*κ* = 0.90; 95% CI: 0.76–1.00; 97% agreement across 34 studies), reflecting the structured nature of the assessment instrument [32, 33]. The total risk of bias across the four bias domains was calculated as follows: 1–1.66 (L), 1.67–2.33 (M), and 2.34–3 (H).

Data were extracted by two experts: the first expert extracted the information, and the second expert reviewed it (Additional file 3). Conflicts were solved by consulting a third reviewer. When information regarding the age or weight of the study animals was lacking, from otherwise eligible references, the corresponding authors were contacted by email for more information. Similarly, the corresponding authors were contacted if extractable data on relevant markers were not reported. Information on additional data provided through personal communication is summarized in Additional file 1: Supplementary Table 6. The data were extracted as the mean and standard deviation (SD) and reported as follows: mg/dL for lipid parameters; % for ejection fraction (EF), fractional shortening (FS), and vasorelaxation; mm for left ventricular internal diameter (LVID), interventricular septum thickness (IVS), and left ventricular posterior wall thickness at diastole (LVPWd); mm/mg for left ventricular mass to body weight ratio (LV/BW). Meta-analysis using a random-effects model was performed for markers when extractable data were available for at least 50% of the relevant studies and with a minimum of two studies included in the analysis. For the remaining markers, a narrative synthesis was performed by following established frameworks [41, 42] involving systematic tabulation, pattern identification, and structured interpretation of mechanistic relationships across studies. Heterogeneity was reported using *I*^2^ statistics, where *I*^2^ > 75% suggested substantial heterogeneity. Subgroup analysis was performed based on intervention type (genetic model or environmental model). The results from meta-analysis were reported in forest plots as standardized mean differences (SMDs) with 95% CIs to account for variations in measurement scales and units across studies [43]. Effect sizes were interpreted according to established conventions: SMD < 0.2 indicating negligible effect, 0.2–0.5 representing small effect, 0.5–0.8 indicating moderate effect, and SMD > 0.8 reflecting large effect [44]. Positive SMDs indicate increased values in circadian-disrupted animals compared to controls, while negative SMDs indicate decreased values. Publication bias was evaluated by funnel plots for markers with more than three studies. Owing to the limited number of studies (*n* < 10 per analysis), statistical tests such as Egger’s test or Begg’s rank correlation were not applied. Instead, funnel plots were visually inspected to assess potential asymmetry; however, this approach is subjective and should be interpreted with caution given the small study numbers. Sensitivity analysis was performed by study removal to identify potentially influential studies. In this review, a modified GRADE (Grading of Recommendations Assessment, Development and Evaluation) approach was used to assess the certainty of evidence for markers for which at least three independent studies reported findings. The GRADE domains were adapted as follows: (1) Risk of bias incorporated the comprehensive four-domain assessment described above, with downgrading if the majority of included studies had moderate or high overall bias scores; (2) Inconsistency was evaluated based on heterogeneity (*I*^2^ statistics) and variation in effect directions across studies, not explained by subgroup or sensitivity analyses; (3) Imprecision was assessed considering total sample sizes, confidence interval width, and whether estimates crossed the null; (4) Indirectness was evaluated based on the relevance of animal models to human shift work scenarios, with consideration of whether findings relied predominantly on genetic models or environmental interventions that better recapitulate intermittent circadian misalignment; and (5) Publication bias was assessed through visual inspection of funnel plots. Evidence certainty was classified as high, moderate, low, or very low. The meta-analyses were performed in R version 4.4.1 (2024–06–14 ucrt) using the meta package (version 8.0–2).

## Results

### Study selection, risk of bias assessment, and study characteristics

The PRISMA flowchart of the included studies is provided in Fig. 1. The systematic search resulted in 9012 references for title and abstract screening after the removal of duplicates. A total of 1148 references passed the pre-screening stage and were subjected to full-text screening. After full-text screening, 34 studies were included in the review and assessed for bias in study design and reporting by the ToxRTool. Of these, two studies were considered to have a high risk of bias, category 3 in the ToxRTool assessment and scored down on the critical questions #11 Appropriate controls [45] and #17 Appropriate study design [45, 46] and were excluded. Of the remaining 32 studies, assessment of bias in study design and reporting (modified ToxRTool), study population, intervention, and analysis were combined into an overall study bias score. Accordingly, 18 studies (56.3%) were classified as having a low risk of bias (L), while 14 studies (43.7%) were classified as having a moderate risk of bias (M). None of the remaining 32 studies was classified as having a high risk of bias in this comprehensive overall assessment. Most studies had low risk of bias in terms of study design and reporting (26 studies, 81.3%) and population bias (29 studies, 90.6%), whereas intervention bias and analytical bias were more frequently rated as moderate. Complete risk of bias assessments for individual studies is presented in Additional file 1: Supplementary Table 7.

The characteristics of the 32 studies included in the synthesis are summarized in Table 1. Among the included studies, 81.3% used mouse models while the remaining studies involved rats (18.8%). C57BL/6J was the most common mouse strain (66.7%), followed by FVB/N (7.4%), ICR (3.7%), and crossbred (11.1%), while some studies did not specify the strain (11.1%). In rat studies, Sprague-Dawley was the most commonly used strain (50.0%), followed by Wistar (33.3%) and outbred white rats (16.7%). Most experiments used male animals (65.5%), while female animals were used in only 3.1% of studies. A smaller number of studies (15.6%) included both sexes, and 15.6% of studies did not specify the sex of the animals. The age of the animals at the beginning of the experiment varied. Half of the studies used animals aged 8–12 weeks, followed by 13–16 weeks (25%) and over 16 weeks (21.9%). Age was not reported in 3.1% of the studies. For these studies, animal weight was used as a proxy for estimation of age. Body weight was reported in 30.8% of the mouse studies and 83.3% of the rat studies. The remaining studies did not specify weight.

**Table 1:** Summary of study characteristics

Characteristics	*N *(%)
Species	
Mouse	26 (81.3%)
C57BL/6	18 (66.7%)
FVB/N	2 (7.4%)
ICR	1 (3.7%)
Crossbreed	3 (11.1%)
NS	3 (11.1%)
Rat	6 (18.8%)
Sprague–Dawley	3 (50.0%)
Wistar	2 (33.3%)
Outbred white	1 (16.7%)
Sex	
Male	21 (65.6%)
Female	1 (3.1%)
Both	5 (15.6%)
NS	5 (15.6%)
Age at beginning of experiment	
8–12 weeks	16 (50.0%)
13–16 weeks	8 (25.0%)
> 16	7 (21.9%)
NS	1 (3.1%)
Body weight at beginning of experiment	
Mice (20–35 g)	8 (30.8%)
Mice (NS)	18 (69.2%)
Rats (200–350 g)	5 (83.3%)
Rats (NS)	1 (16.7%)
Experimental model	
Genetic intervention	21 (63.6%)
Environmental intervention	12 (36.4%)
Effect outcome	
Cardiac	14 (30.0%)
Vascular	19 (40.0%)
Cardiovascular	14 (30.0%)

Two main types of interventions were identified among the experimental models. In 63.6% of the studies, circadian rhythm was modified using circadian gene mutants or KO models. Additionally, environmental intervention models (36.4%), such as light phase shift or sleep fragmentation models, were used. Detailed information on the study descriptions can be found in Additional file 1: Supplementary Table 8.

The extracted effect outcomes were grouped into cardiac effects, vascular effects, and cardiovascular effects, where 30% of the studies reported cardiac effects, 40% of the studies reported vascular effects, and 30% of the studies reported cardiovascular effects. The key findings are summarized in Additional file 1: Supplementary Table 9.

### Synthesis

#### Effects of circadian disruption on cardiac markers

Cardiac dysfunction was stratified into four domains: (1) impairment of excitability; (2) impairment of contractility/relaxation; (3) cardiomyocyte injury and death; and (4) dysregulation of valve stroma. Of the 14 studies identified, only one investigated the impairment of excitability, while 12 investigated the impairment of contractility/relaxation, and 10 investigated cardiomyocyte injury and death. No studies have investigated the dysregulation of valve stroma proliferation. The experimental models used for investigating cardiac dysfunction were mostly genetically modified models (64.3%), whereas 35.7% of the studies implemented environmental interventions.

##### Cardiac excitability

The search identified only one study on the role of the circadian clock in cardiac excitability. In this study, a cardiomyocyte-specific *Bmal1* KO (iCSΔ*Bmal1* − / −) mouse model was used to explore K⁺ channel expression and ventricular repolarization [47]. They reported that *Kcnd2* and *Kcnh2* transcripts exhibited circadian oscillations in hearts of WT mice, but the circadian rhythm of *Kcnh2* was lost in *Bmal1* KO mice. Moreover, the expression of non-circadian potassium channel transcripts (*Kcnip2*, *Kcna5*, *Kcnb1*, *Kcnj2*, and *Kcnq1*) was reduced in hearts of *Bmal1* KO mice. These molecular changes were associated with prolonged heart rate-corrected QT intervals (QTc) during the light (rest) phase.

##### Impairment of contractility/relaxation

Twelve studies investigated the impact of circadian disruption on cardiac contractility and relaxation. The most reported biomarkers were ejection fraction (EF), fractional shortening (FS), and left ventricular internal diameter (LVID).

**Fig. 2:**
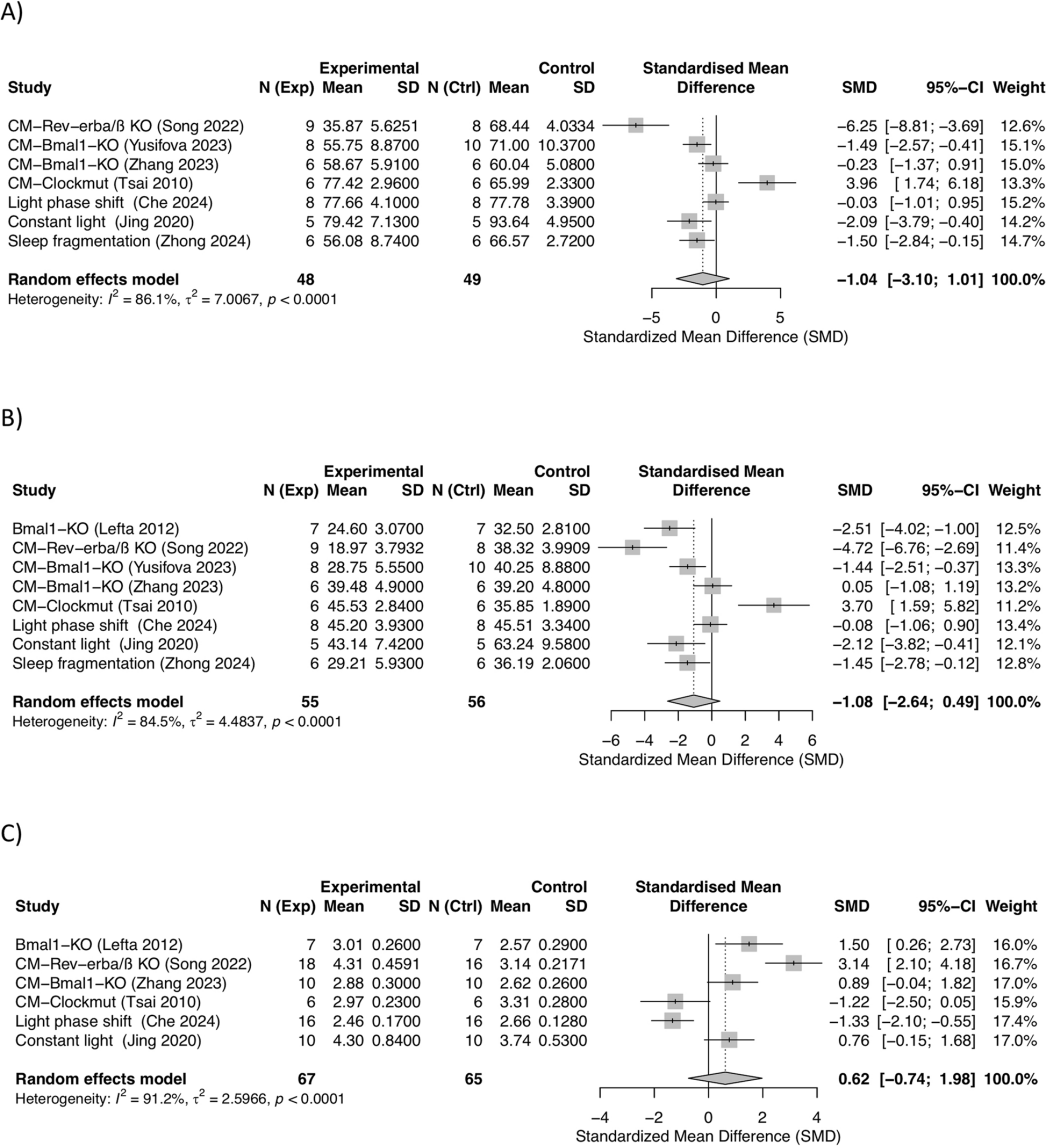
Meta-analyses evaluating the effects of circadian disruption on cardiac contractility and relaxation across intervention models. **A** Ejection fraction (EF), **B** fractional shortening (FS), and **C** left ventricular internal diameter (LVID) were analyzed across genetic and environmental models. Data are presented as standardized mean differences (SMDs) with 95% CI.

To quantitatively assess the effects of circadian disruption on EF (*n* = 7/8), FS (*n* = 8/9), and LVID (*n* = 6/7), meta-analysis was performed. The overall meta-analysis revealed no significant effect on EF (SMD: − 1.04, 95% CI: − 3.10 to 1.01; *I*^2^ = 86.1%), FS (SMD: − 1.08, 95% CI: − 2.64 to 0.49; *I*^2^ = 84.5%), and LVID (SMD: 0.62, 95% CI: − 0.74 to 1.98; *I*^2^ = 91.2%) (Fig. 2A–C). However, the *I*^2^ statistics indicated very high heterogeneity among studies. Funnel plots revealed an overall symmetrical distribution with some heterogeneity, as two studies fell outside the 95% confidence limits, suggesting limited evidence of publication bias (Additional file 1: Supplementary Fig. S1A–C).

Leave-one-out sensitivity analysis revealed that the overall estimates of EF and FS were most sensitive to the study by Tsai et al. (Additional file 1: Supplementary Fig. S2A, B), which investigated the effects of a functional mutation in the *Clock* gene in a *Clock* mutant (*Clockmut*) mouse model and demonstrated increased EF and FS compared with those of wild-type (WT) controls [48]. Omitting this study decreased the observed heterogeneity of both EF and FS markers, and the sensitivity analysis showed a significantly large reduction in EF (SMD: − 1.70, 95% CI: − 3.22 to − 0.17; *p* = 0.0290, *I*^2^ = 79.6%) and FS (SMD: − 1.60, 95% CI: − 2.71 to − 0.49; *p* = 0.0047, *I*^2^ = 76.6%) following circadian disruption. These findings suggest that effects of the *Clockmut* model on these two cardiac markers may differ substantially, influencing both the magnitude and direction of the pooled effects. However, while omitting the study by Tsai et al. [48] reduced the heterogeneity for EF and FS, the *I*^2^ yet remained above 75% for both markers. For LVID, no clear outliers were identified by leave-out-one sensitivity analysis (Additional file 1: Supplementary Fig. S2C).

To further explore the underlying reasons for the observed heterogeneity, subgroup analysis by intervention model, i.e., genetic and environmental, was performed (Additional file 1: Supplementary Fig. S3). Subgroup analysis showed that the genetic models had a very high heterogeneity (*I*^2^ = 92%, *I*^2^ = 89.9%, and *I*^2^ = 89.2% for EF, FS, and LVID, respectively). Thus, although a trend toward reduced levels of EF and FS is present in KO models, great care should be taken in the interpretation of these findings. Most studies reported reduced EF and FS in global [19, 49] and cardiomyocyte-specific *Bmal1* KO mice [16]; however, one study utilizing a cardiomyocyte-specific *Bmal1* KO model reported no significant changes in EF or FS compared with WT controls [50]. Moreover, cardiac-specific *Nr1d1/Nr1d2* double KO mice showed progressive contractile dysfunction, with normal EF and FS values at 2.5 months but 20–30% declines by 6 months [17]. For the environmental models, no effect on EF and FS was observed, and the relatively low heterogeneity suggests greater concordance in observed effects from the environmental models.

Additional data on other functional parameters might indicate altered cardiac relaxation upon circadian disruption. Zhang et al. reported a significant reduction in the E/A ratio and increases in isovolumetric relaxation time (IVRT) and E/e′ ratio in cardiomyocyte-specific *Bmal1* KO mice, indicating diastolic dysfunction [50]. Similarly, a significant decrease in E/A ratio was observed in a light-induced rolling phase shift model [51]. Constant light exposure to rats reduced + dP/dT, providing direct evidence of weakened contractile force [21]. Furthermore, SERCA2 protein levels, critical for calcium reuptake during diastole, showed model-dependent and time-dependent alterations, as it was increased in shift work-simulated mice but reduced in *Clockmut* mice [18] and, moreover, progressively decreased with a significant reduction in SERCA expression observed after 5 weeks of dim light exposure [52]. Altogether these findings indicate that impaired calcium transport may develop gradually following prolonged circadian disruption.

##### Cardiomyocyte injury and death

Among the 10 studies investigating the effects of circadian disruption on cardiomyocyte injury and death, the most reported biomarkers were cardiomyocyte size, left ventricular mass to body weight ratio (LV/BW), left ventricular posterior wall thickness at diastole (LVPWd), and interventricular septum thickness (IVS). Other biomarkers, such as apoptosis markers, natriuretic peptides, fibrosis markers, and dystrophin expression, were reported less frequently and may serve as emerging biomarkers of cardiac dysfunction under circadian misalignment.

**Fig. 3:**
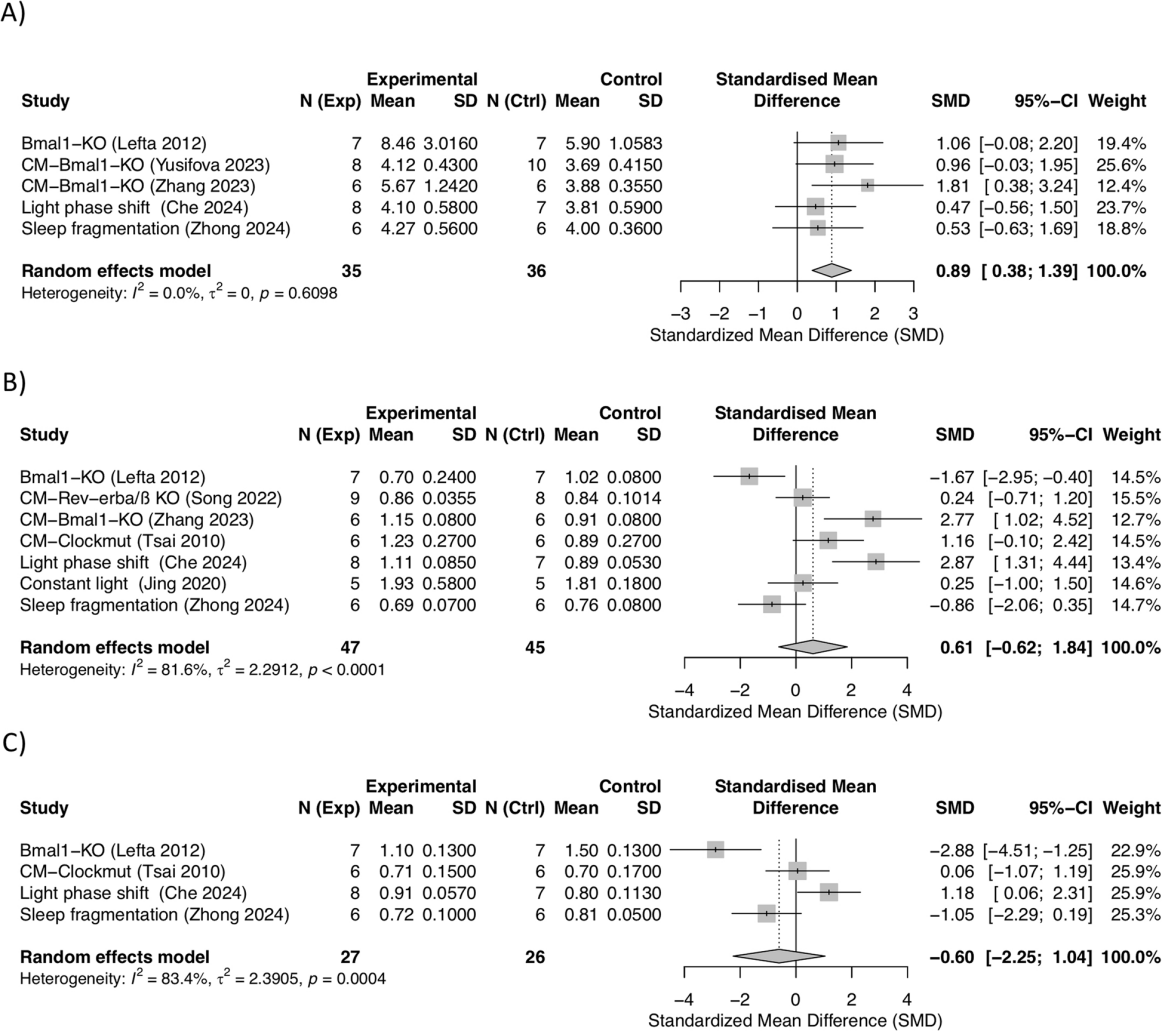
Meta-analyses by intervention model of structural indicators of cardiomyocyte injury and death **A** left ventricular mass to body weight ratio (LV/BW), **B** left ventricular posterior wall thickness at diastole (LVPWd), and **C** interventricular septum thickness (IVS), under circadian disruption. Data are presented as standardized mean differences (SMDs) with 95% CI

Meta-analysis was performed for LV/BW (*n* = 5/5), LVPWd (*n* = 7/8), and IVS (*n* = 4/5). LV/BW was significantly increased across the models (SMD: 0.89, 95% CI: 0.38 to 1.39; *I*^2^ = 0%), Cardiac hypertrophy, assessed by left ventricular mass to body weight ratio (LV/BW), was significantly increased in circadian-disrupted animals compared to controls across 12 studies (SMD = 0.85, 95% CI 0.62–1.08, *p* < 0.001; *I*^2^ = 67%, Fig. 3A), indicating a large effect size (Fig. 3A). The data showed low heterogeneity and no publication bias (Additional file 1: Supplementary Fig. S4A). Sensitivity analysis confirmed the robustness of the results, with findings remaining consistent across all leave-one-out scenarios (Additional file 1: Supplementary Fig. S5A). Furthermore, subgroup analysis showed that the effect originated predominantly from genetic models (SMD: 1.18, 95% CI: 0.51 to 1.84; *I*^2^ = 0%) (Additional file 1: Supplementary Fig. S6A), which included both global *Bmal1* KO [49] and cardiomyocyte-specific *Bmal1* KO models [16, 50] (Fig. 3A). No effect was observed on the environmental models. Meta-analyses on LVPWd (SMD: 0.61, 95% CI: − 0.62 to 1.84; *I*^2^ = 81.6%) and IVS (SMD: − 0.60, 95% CI: − 2.25 to 1.04; *I*^2^ = 83.4%) demonstrated no significant effect of circadian disruption on these indicators (Fig. 3B and C). However, for both indicators, the *I*^2^ statistics indicated high heterogeneity with considerable spread of the data, but funnel plots showed no clear evidence of publication bias (Additional file 1: Supplementary Fig. S4B and C). For LVPWd, decreased thickness was reported in global *Bmal1* KO [19, 41], while increased thickness was seen in cardiomyocyte-specific *Bmal1* KO [46] and light phase shift-based jet lag model [22]. No change was observed in *Clockmut* [38], constant light [21], or sleep fragmentation [24]. For IVS, reductions were observed in global *Bmal1* KO models [19, 49], whereas *Clockmut* [48], sleep fragmentation [24], and the light phase shift-based jet lag model [22] showed either no effect or slight increases. Sensitivity analysis did not find any single study that substantially influenced the effect estimates for LVPWd and IVS (Additional file 1: Supplementary Fig. S5B and C), supporting the robustness of the findings and the lack of true effect for these markers.

Due to methodological heterogeneity and limited quantitative reporting across studies, meta-analysis of cardiomyocyte size was not feasible. Five studies reporting this outcome employed different measurement approaches, including histological staining (wheat germ agglutinin, lectin) and direct measurement of isolated cardiomyocytes, with results reported in different units (μm^2^, pixels). Importantly, only one study provided numerical data, whereas the remaining studies presented results exclusively as images without quantitative values. Despite these limitations, cardiomyocyte size was significantly increased in four studies, including cardiomyocyte-specific *Clockmut* mice [18], global *Bmal1* KO [19], cardiomyocyte-specific *Bmal1* KO [16], and chronic jet lag models [22]. On the contrary, Lefta et al., using global *Bmal1* KO, showed that the average cardiomyocyte size decreased: 19% decrease (*Bmal1* KO 2538 ± 470.88 vs. wild type 3152 ± 796 μm^2^, not significant) in *Bmal1* KO hearts, despite observing cardiac enlargement [45]. Further investigation by Lefta et al. revealed a significant shift in cardiomyocyte size distribution, with a greater proportion of small cardiomyocytes (< 2500 μm^2^) and fewer large cardiomyocytes in *Bmal1* KO mice. The authors suggested that observed cardiac hypertrophy may have resulted from increased cell number (hyperplasia) or non-cardiomyocyte tissue expansion rather than from the hypertrophy of individual cardiomyocytes. However, the predominant finding of increased cardiomyocyte size across diverse circadian disruption models indicates that cardiac hypertrophy is a robust response to circadian dysfunction. Increased cardiomyocyte size is a canonical histological marker of pathological hypertrophy in preclinical models and aligns with elevated LV/BW and molecular hypertrophy markers observed in several models [53].

Additional studies have reported effects on other cellular and molecular markers related to cardiomyocyte injury in circadian-disrupted hearts. Natriuretic peptides (ANP, BNP) levels were consistently elevated in cardiomyocyte-specific *Bmal1* KO [16] and cardiomyocyte-specific *Nr1d1/Nr1d2* KO models [17], supporting their role as convergent markers of hypertrophic signaling under circadian disruption. Durgan et al. reported increased *Nppa* and *Rcan1* and decreased *Myh6* mRNA expressions in *Clockmut* models, indicating cardiac hypertrophy [18]. Structural protein alterations included reduced dystrophin expression in cardiomyocyte-specific *Bmal1* KO mice [53] and sarcomere disorganization in global *Bmal1* KO hearts [19], suggesting myocardial structural remodeling. Fibrotic remodeling was evident in both cardiomyocyte-specific *Bmal1* KO [16] and light-based chronic jet lag models [22]. The chronic jet lag model also showed increased mRNA expression of pro-fibrotic markers (*Myh7*, *Lgalsl3*, *Acta2*). Cardiomyocyte-specific *Bmal1* knockdown triggered apoptotic pathway activation, with increased TUNEL-positive nuclei and elevated mitochondrial apoptosis markers (cleaved caspases, cytochrome C, poly (ADP-ribose) polymerase (PARP), and second mitochondria-derived activator of caspases (Smac) [50].

#### Effects of circadian disruption on vascular markers

Studies investigating the effects of circadian disruption on vascular function focused on three key domains: (1) endothelial dysfunction and vascular effects; (2) dysregulation of hemostasis; and (3) dyslipidemia. Among the 19 studies, eight studies examined endothelial dysfunction and vascular remodeling, five studies assessed hemostasis dysregulation, and 11 studies investigated dyslipidemia.

##### Endothelial dysfunction and vascular effects

Of the studies investigating the impact of circadian disruption on endothelial dysfunction, vasorelaxation was the most studied biomarker while other biomarkers were also explored such as vascular wall thickness, collagen deposition, leukocyte rolling, mean arterial pressure, endothelin-1 (ET1), angiotensin type 1 receptor (AT1R), and angiopoietin-like protein 2 (ANGPTL2) expression.

**Fig. 4:**
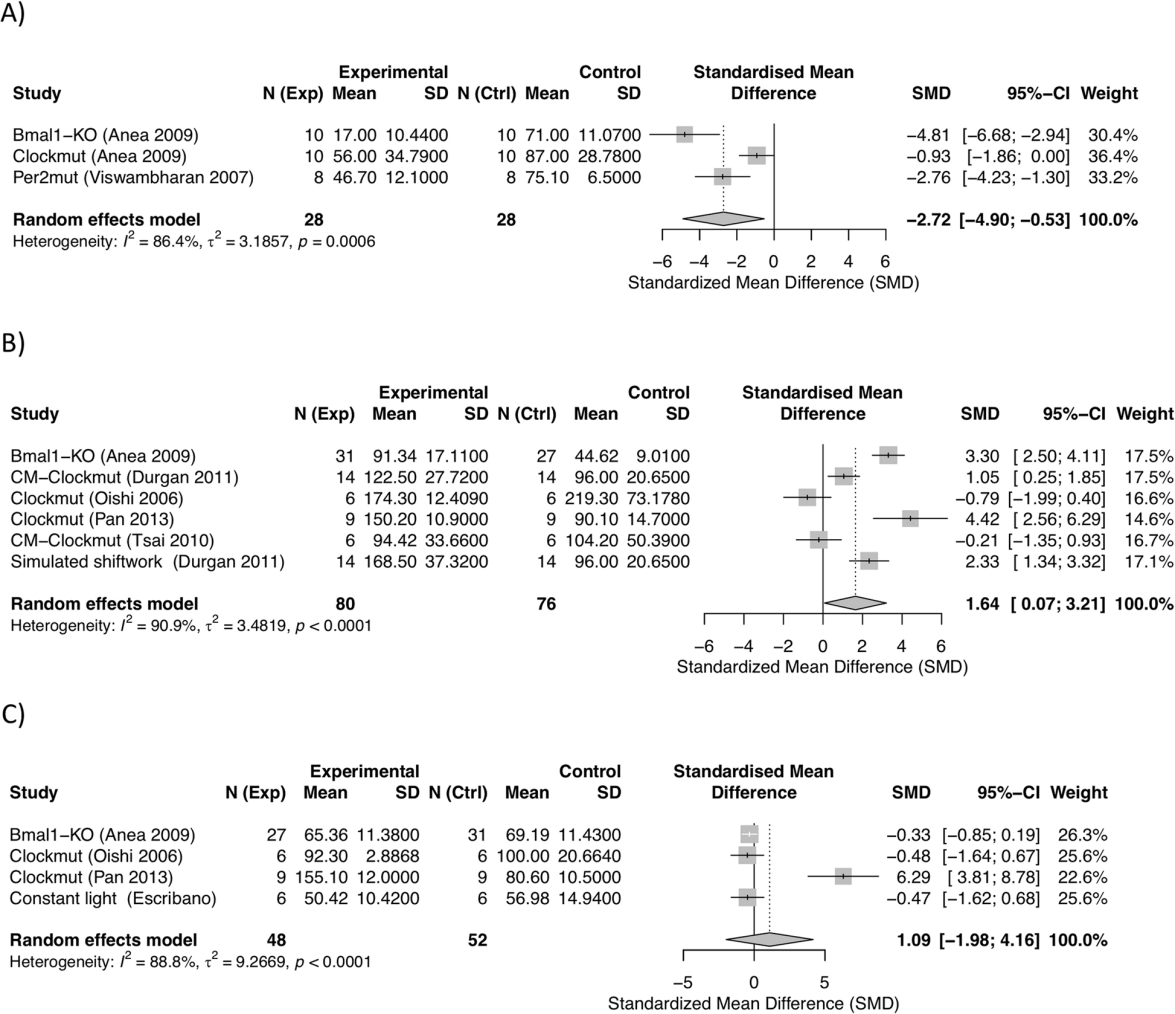
Meta-analyses of intervention models of vascular and lipid biomarkers associated with circadian disruption. **A** Acetylcholine-induced endothelium-dependent vasorelaxation, **B** plasma triglyceride (TG), and **C** total cholesterol (TC) levels were evaluated across genetic and environmental models. Data are presented as standardized mean differences (SMD) with 95% CI

Of four studies reporting vasorelaxation, three studies on genetic models were included in the meta-analysis and showed that circadian disruption results in impaired endothelium-dependent vasorelaxation, with a significant overall reduction in acetylcholine-induced vasorelaxation (SMD: − 2.72, 95% CI: − 4.90 to − 0.53; *I*^2^ = 86.4%), representing a very large effect size (Fig. 4A). The limited number of studies reporting data and the high heterogeneity imply that the findings need to be interpreted with caution. Moreover, no significant effects on acetylcholine-induced vasorelaxation or smooth muscle contraction were reported in an additional study in *Bmal1* KO mice [54], suggesting that circadian disruption likely does not directly affect vascular contractility.

In addition to vasorelaxation, the effects of circadian disruption on structural vascular remodeling have been reported. Accordingly, *Bmal1* KO mice demonstrated abnormal vascular adaptation following carotid ligation and increased collagen deposition in arterial walls, suggesting increased vascular stiffness [55]. Moreover, *Cry1/2* KO mice housed under constant darkness for 36-h lost rhythmic ANGPTL2 expression, implicating circadian regulation in vascular repair and inflammation [56]. Environmental circadian disruption models have also been used to investigate effects on vascular parameters, showing that exposure to chronic light may result in increased mean arterial pressure [57]. However, no effects on blood pressure were observed in *Clockmut* mice or in animals exposed to bi-weekly 12-h phase shifts for 16 weeks [18]. Finally, prolonged exposure to dim light at night resulted in reduced ET1 and AT1R protein levels in the left ventricle, suggesting early changes in vascular responsiveness [52].

##### Dysregulation of hemostasis

Several studies were identified that supported the role of circadian disruption on hemostatic parameters. Studies have shown increased thrombus formation, elevated levels of fibrinogen and plasminogen activator inhibitor-1 (PAI1), and shortened arteriolar and venular occlusion times, which despite prolonged activated partial thromboplastin time (aPTT), collectively supporting a shift toward a pro-thrombotic state in *Bmal1* KO mice [54, 55]. Moreover, the presence of an unaffected platelet activation marker (CD41) may indicate that platelet activation is likely not the primary driver of the thrombotic phenotype. *Clock* knockdown models also showed reduced levels of activated protein C, a key anti-coagulant, further supporting the role of circadian regulation in maintaining hemostatic balance [58]. Finally, impaired platelet aggregation and prolonged bleeding times, but unchanged platelet counts and clotting parameters, were reported in *Nr1d1* KO mice [59], indicating that *Nr1d1* primarily regulates platelet activation rather than overall coagulation cascade components. Moreover, environmental effectors of circadian rhythm such as prolonged light exposure (18:6 LD model) significantly increased platelet aggregation parameters at 10 days, but by 21 days, aggregation efficiency decreased [60], suggesting that acute and chronic circadian misalignment may have opposing effects on thrombotic potential.

##### Dyslipidemia

Studies related to lipid metabolism reported findings predominantly on triglyceride (TG) and total cholesterol (TC) levels, for which meta-analyses were performed. For TG, meta-analysis was performed on 6 out of 10 studies, showing overall elevated TG levels following circadian disruption (SMD: 1.64, 95% CI: 0.07 to 3.21; *I*^2^ = 90.9%), indicating a large effect size, although with high heterogeneity (Fig. 4B). No publication bias was identified (Additional file 1: Supplementary Fig. S7). Sensitivity analysis by study removal did not reduce the observed heterogeneity (Additional file 1: Supplementary Fig. S8). Importantly, within the studies lacking extractable data and thus not included in the meta-analysis, three studies showed no effects [61–63] and one study showed decreased TG levels [23]. For TC, meta-analysis of four studies demonstrated no effect of circadian disruption on this marker (SMD: 3 1.09, 95% CI: − 1.98 to 4.16; *I*^2^ = 88.8%) (Fig. 4C, Additional file 1: Supplementary Fig. S7).

In addition, despite elevated levels of high-density lipoprotein (HDL) cholesterol, *Clockmut* mice exhibit an increase in apoB-lipoprotein cholesterol and pro-atherosclerotic phenotype with increased plaque formation and lipid lesions [20]. Moreover, chronic circadian disruption through repeated phase shifts selectively increased low-density lipoprotein cholesterol, whereas other lipid parameters remained unchanged [63]. Finally, Mia et al. examined TG synthesis specifically within the heart and found no significant differences between cardiomyocyte-specific *Bmal1* KO and WT mice [64], suggesting that BMAL1 may potentially influence circulating TG levels but does not regulate TG synthesis within cardiac tissue.

#### Effects of circadian disruption on cardiovascular markers

Five key domains, namely, (1) mitochondrial dysfunction, (2) modification of autonomic nervous system (ANS) activity, (3) oxidative stress, (4) inflammation, and (5) alteration of hormone signaling, were investigated for cardiovascular effects. Of the 14 studies identified in the literature search, two reported biomarkers of mitochondrial dysfunction, four reported biomarkers of ANS activity, seven reported oxidative stress, and four reported inflammation markers. No studies on circadian disruption-mediated alteration of hormone signaling were identified.

##### Mitochondrial dysfunction

Only two eligible studies investigating the impact of circadian disruption on mitochondrial function in the cardiovascular system were identified in the literature search. Mia et al. examined the effects of cardiomyocyte-specific *Bmal1* KO in mice and assessed mitochondrial complex protein levels and enzymatic activity in the heart [64]. While no significant differences were observed in the levels or activities of mitochondrial complexes I, II, and III, a slight reduction in complex II levels and a moderate increase in complex IV levels were noted in the hearts of the KO mice compared with those of the WT controls. Assessment of mitochondrial enzyme activity showed increased complex IV activity, though the change was not statistically significant. These findings indicate that cardiomyocyte-specific deletion of *Bmal1* does not induce major mitochondrial dysfunction in the heart, suggesting that mitochondrial oxidative phosphorylation may be less sensitive to circadian disruption in this model. A second study explored the impact of light exposure on mitochondrial redox balance in Wistar rats, where the animals were subjected to either constant light (24-h L) or complete darkness (22-h D) for 2 weeks [65]. The study found that total glutathione (GLT), reduced glutathione (GSH), catalase (CAT), glutathione peroxidase (GPx), glutathione reductase (GRd), and glutathione transferase (GST) were significantly reduced in the hearts of rats exposed to constant light, indicating a weakened mitochondrial antioxidant defense system. Conversely, rats kept in constant darkness exhibited increased GSH, GRd, GST, and CAT levels, suggesting enhanced mitochondrial protection in the absence of light-induced circadian disruption.

##### Dysregulation of ANS

Increased norepinephrine levels were reported in two studies. Accordingly, plasma norepinephrine levels were significantly increased (118.3 ± 4.4 ng/ml vs. 142.6 ± 3.4 ng/ml), in a light phase shift model where light onset was advanced by 6-h every 2 days for 42 days [51]. Similarly, animals exposed to constant light had increased plasma norepinephrine levels compared to those housed in a normal 12:12 light–dark cycle, suggesting that chronic light exposure upregulates sympathetic nervous system activity [21]. Furthermore, inducible *Bmal1* KO mice under constant darkness lost urinary norepinephrine rhythmicity [66]. Light re-entrainment did not restore this rhythm, supporting the importance of endogenous clocks in regulating norepinephrine dynamics. On the contrary, Sutovska et al. examined tyrosine hydroxylase expression in rats exposed to artificial light at night for 2 or 5 weeks and found no significant changes, suggesting that tyrosine hydroxylase, a rate-limiting enzyme in catecholamine biosynthesis, remains stable despite circadian misalignment [48].

##### Oxidative stress

The search identified seven studies assessing oxidative stress in circadian disruption models by measuring markers of reactive oxygen species (ROS) and lipid peroxidation. Reduced levels of phosphorylated nitric oxide synthase 3 (NOS3) were found in *Bmal1* KO mice, suggesting compromised nitric oxide signaling [55]. Similarly, mice subjected to 8-h advances in the light period every 4 days for 3 months showed decreased cardiac NOS3 protein levels [22]. Furthermore, the expression of NADPH oxidase 4 (*Nox4*) increased 3.5-fold in the aortas of *Bmal1* KO mice, leading to increased hydrogen peroxide production in endothelial cells [67]. *Per2* mutant mice also exhibited increased cyclooxygenase 1 (*Cox1*) expression in aortic tissue, reinforcing the link between circadian genes and oxidative stress regulation [62]. In a light intervention model, Escribano et al. showed that continuous light led to increased lipid peroxidation, whereas continuous darkness led to reduced lipid peroxidation [65]. Moreover, sleep disruption resulted in reduced serum antioxidant levels, further confirming that circadian misalignment may promote oxidative damage by reducing antioxidant capacity [68].

##### Inflammation

Studies investigating the relationship between circadian disruption and inflammation assessed various biomarkers, including leukocyte counts, inflammatory cytokines, chemokines, C-reactive protein (CRP), and neutrophil extracellular trap (NET) formation. These findings indicate that circadian disruption may induce systemic and cardiac inflammation in multiple models. Hemmeryckx et al. found that *Bmal1* KO mice exhibited increased leukocyte and neutrophil counts, with slightly elevated CRP levels, indicating a pro-inflammatory state [54]. Similarly, the mRNA expression levels of chemokine ligand 2 (*Ccl2*) and chemokine receptor types 2 and 5 (*Ccr2* and *Ccr5*) were upregulated in cardiac-specific *Nr1d1/Nr1d2* KO mice at 4.5 months, whereas interleukin 1 beta (*Il1b*) and tumor necrosis factor (*Tnf*) mRNA levels remained unchanged [17]. Furthermore, increased IL1B, interleukin 6 (IL6), and TNF protein levels were observed in sleep disrupted mice [68]. Finally, using a light-induced circadian rhythm disorder model, Wang et al. demonstrated that circulating neutrophils presented increased levels of NET formation, as indicated by increased cell-free DNA and myeloperoxidase levels, suggesting increased neutrophil activation and increased vascular inflammation [51].

### Certainty of evidence and consistency of findings

Based on a modified GRADE approach, the certainty of evidence for biomarkers with more than three available references was assessed (Additional file 1: Supplementary Table 10). The evidence for associations between circadian disruption and markers of cardiac, vascular, and cardiovascular function is summarized in Table 2.

**Table 2:** Overall associations between circadian disruption and the assessed cardiovascular risk markers

Biomarker	No. of animals (studies)	Estimated effect	Certainty of evidence	Interpretation
Cardiomyocyte size	68 (5)	Four studies reported an increase while one study showed no effect	⊕ ⊕ ⊕ ◯ Moderate	Cardiomyocyte size increased consistently through most studies; however, certainty was moderate due to lack of quantitative synthesis
EF	97 (7)	SMD: − 1.04 [− 3.10, 1.01]; sensitivity analysis: SMD: − 1.70 [− 3.22, − 0.17]	⊕ ⊕ ◯◯ Low	Circadian disruption may reduce EF, with a large effect seen in sensitivity analysis; however, the main analysis was imprecise and not statistically significant, leading to uncertainty in the true effect
FS	111 (8)	SMD: − 1.08 [− 2.64, 0.49]; sensitivity analysis: SMD: − 1.60 [− 2.71, − 0.49]	⊕ ⊕ ◯◯ Low	Circadian disruption may reduce FS, with a large effect seen in sensitivity analysis; however, the main analysis was imprecise and not statistically significant, leading to uncertainty in the true effect
IVS	53 (4)	SMD: − 0.60 [− 2.25, 1.04]	⊕ ⊕ ◯◯ Low	Circadian disruption did not affect IVS, but the evidence is uncertain due to inconsistency and imprecision in the effect estimate
LV/BW	71 (5)	SMD: 0.89 [0.38, 1.39]	⊕ ⊕ ⊕ ⊕ High	High-certainty evidence indicates that circadian disruption increases LV/BW, with a large effect size
LVID	132 (6)	SMD: 0.62 [− 0.74, 1.98]	⊕ ◯◯◯ Very low	Circadian disruption did not affect LVID, but the evidence is uncertain due to inconsistency and imprecision in the effect estimate
LVPWd	92 (7)	SMD: 0.61 [− 0.62, 1.84]	⊕ ◯◯◯ Very low	Circadian disruption did not affect LVPWd, but the evidence is uncertain due to inconsistency and imprecision in the effect estimate
TC	100 (4)	SMD = 1.09 [− 1.98, 4.16]	⊕ ⊕ ◯◯ Low	Circadian disruption did not affect TC, but the evidence is uncertain due to inconsistency and imprecision in the effect estimate
TG	156 (6)	SMD = 1.64 [0.07, 3.21]	⊕ ⊕ ◯◯ Low	Circadian disruption may increase TG, but the evidence is uncertain due to inconsistency and imprecision in the effect estimate
Vasorelaxation	56 (3)	SMD = − 2.72 [− 4.90, − 0.53]	⊕ ⊕ ◯◯ Low	Circadian disruption may substantially reduce vasorelaxation, with a very large effect size. However, the evidence is uncertain due to high heterogeneity and limited number of studies

Evidence suggests that circadian disruption may result in cardiac hypertrophy at the organ level. Accordingly, high-certainty evidence indicates a large increase in the LV/BW ratio, suggesting significant left ventricular hypertrophy in animal models of circadian disruption. Moreover, narrative synthesis by vote count found moderate evidence for cardiomyocyte hypertrophy as although cardiomyocyte size was consistently increased in all models of circadian disruption, the evidence was limited by the lack of quantitative synthesis. For both EF and FS, no significant effect was observed in the main analysis; however, sensitivity analysis reduced the heterogeneity resulting in a large significant reduction in these indicators in animal models of circadian disruption, suggesting the presence of poor systolic function and impaired contractility. For other cardiac indicators, i.e., LVID, IVS, and LVPWd, small-to-no effects were observed following circadian disruption at very low-to-low certainty.

For vascular function, low-certainty evidence suggests that circadian disruption may result in substantially impaired vasorelaxation. For markers of dyslipidemia, low-certainty evidence suggests increased TG levels in animal models of circadian disruption. For TC, no effects were observed, but the high uncertainty of evidence warrants further investigation.

Cardiovascular biomarkers related to mitochondrial dysfunction, ANS activity, oxidative stress, inflammation, and hormone signaling alterations did not meet the three-study inclusion threshold and the certainty of evidence was not graded. However, individual findings suggest mechanistic links between circadian disruption and cardiovascular risk, highlighting the need for further research.

## Discussion

### Summary of findings and implications

To our knowledge, this review is the first to systematically synthesize and grade the existing in vivo mechanistic evidence on the effects of circadian disruption, defined as models of circadian gene modifications, light interventions, and sleep deprivation, on cardiac, vascular, and cardiovascular indicators of cardiovascular risk.

The synthesis provided evidence of increased left ventricular mass-to-body weight ratio (LV/BW) with high certainty and increased cardiomyocyte size with moderate certainty in animal models of circadian disruption. Individual reports also showed increased levels of ANP, BNP, and RCAN1, as well as reduced levels of α-MHC. Natriuretic peptides (i.e., ANP and BNP) levels are increased following hemodynamic stress in the ventricles [69]. Furthermore, elevated RCAN1 expression together with decreased α-MHC levels indicate pathological remodeling and hypertrophic signaling activation [69]. Altogether, these indicators support the occurrence of ventricular wall thickening through either cardiomyocyte hypertrophy or fibrosis. In further support, the meta-analysis showed reduced EF and FS in circadian disruption models, with the exception of *Clockmut* mice, which seemingly had substantially different effects on these two cardiac markers influencing both the magnitude and the direction of the pooled effects. While the evidence for EF and FS was downgraded due to inconsistency and high heterogeneity, sensitivity analysis by removal of the *Clockmut* model resulted in a significant reduction in these markers. Moreover, individual studies also reported evidence of increased expression of pro-fibrotic markers and fibrotic remodeling in animals with disrupted circadian rhythm. Altogether, these reports suggest the presence of pathological cardiac hypertrophy in animal models of circadian disruption, with potential translational implications for shift-worker health. However, a recent meta-analysis of cardiovascular indicators in night shift workers [27] identified only one study on left ventricular hypertrophy [70] and one on NT-proBNP levels [71], both studies demonstrating no significant associations with night shift work. Identification of only two studies investigating the effect of night shiftwork on cardiac hypertrophy indicates a critical knowledge gap in occupational epidemiology. While elevated BNP or NT-proBNP levels are used as diagnostic and prognostic markers for hypertrophy and heart failure, these remain substantially underexplored in occupational studies. Future epidemiological studies on night shift workers would benefit from incorporating echocardiographic assessment of left ventricular mass alongside circulating natriuretic peptides (BNP, NT-proBNP) and cardiac troponins to detect early signs of circadian disruption-induced cardiac remodeling.

Evidence for a possible effect of night shift work on cardiac excitability, with prolonged QTc among night shift workers, has been reported in a recent systematic review of epidemiological cardiovascular risk indicators [27]. Additionally, one study reported prolonged QTc intervals along with decreased potassium channel gene expression in cardiomyocyte-specific *Bmal1* KO mice [47]. These findings emphasize the potential importance of cardiac excitability as a risk indicator in circadian misalignment, warranting further research.

An increased risk of hypertension has been extensively reported among both fixed and rotating shift workers [4, 28]. However, in vivo evidence on mechanistic indicators of vascular dysfunction was found inconclusive in this review. Accordingly, chronic light exposure may lead to increased mean arterial pressure [57], but no effects on blood pressure were observed in *Clockmut* mice or in animals exposed to extended bi-weekly phase shifts [18]. Furthermore, very low-certainty evidence suggests that circadian disruption may result in substantially impaired vasorelaxation with a large SMD of − 2.72 [− 4.90, − 0.53]. However, these data were based on only three studies, which were all genetic models and should be interpreted with caution. Evidently, genetic disruptions of circadian gene regulation may induce significant endothelial impairment; however, lack of evidence from environmental models downplays the significance of these findings. Together with hypertension, dyslipidemia is an important risk factor for atherosclerosis. It should be noted that murine models are less ideal for studying dyslipidemia and atherosclerosis as both mice and rats have higher HDL levels than humans do, and they do not develop spontaneous atherosclerosis [72]. Elevated TG levels (SMD: 1.64, 95% CI: 0.07 to 3.21) were observed in animal models of circadian disruption. However, extractable data included in this meta-analysis were available from only six of ten studies, whereas the remaining studies reported no or decreased TG levels. Thus, the observed effect is likely overestimated, and together with the high heterogeneity in the data, this evidence is considered to be of low confidence.

Finally, narrative synthesis revealed elevated levels of inflammatory markers, e.g., cytokines, chemokines, and CRP [17, 54, 68], and increased oxidative stress manifested as decreased NOS3 levels [22, 55], increased cyclooxygenase 1 (COX1) expression [62], reduced antioxidant activity [68], and increased lipid peroxidation [65] in animal models of circadian disruption. These findings are consistent with epidemiological evidence from systematic reviews on shift workers, showing increased inflammation, e.g., CRP and leukocyte counts [27], and increased oxidative stress, e.g., increased oxidative stress markers, reduced antioxidant capacity, and increased levels of oxidative DNA damage [73], among night shift workers. Thus, the inflammation and oxidative stress pathways represent important translational targets that warrant further experimental investigation.

### Strengths and limitations

This systematic review provides a comprehensive synthesis of in vivo studies investigating circadian disruption and cardiovascular risk indicators. A major strength is the structured approach to evidence synthesis, including systematic risk of bias assessment, certainty of evidence evaluation, and quantitative meta-analyses where feasible. By integrating findings from both genetic and environmental models, this review highlights potential mechanistic pathways underlying cardiovascular risk. The use of a modified GRADE approach enhances transparency in certainty grading and strengthens the reliability of the conclusions drawn.

Despite these strengths, several limitations should be acknowledged. While this review aimed to provide a comprehensive overview of the current evidence to avoid overlooking relevant mechanistic insights, the included studies displayed substantial heterogeneity in experimental design, including differences in animal strains, circadian disruption methods, and biomarker assessments. Both genetic and environmental models provide complementary insights, but with distinct methodological limitations and varying risks of bias. To address this variability, methodological differences were accounted for in the risk of bias assessment and weight of evidence grading, where each study type was assigned a different weight based on its transferability, reproducibility, and experimental rigor.

In our assessment, tissue-specific genetic interventions (KO and mutant models) were scored as having a low risk of bias, as they provide precise targeting of clock components in cardiovascular tissues with minimal confounding effects. In contrast, global KO models were classified as having a moderate risk of bias due to potential developmental compensation and secondary systemic effects that may obscure direct clock-mediated cardiovascular phenotypes. Among the environmental models, light phase shift interventions were scored as having a low risk of bias, as they better recapitulate the intermittent circadian misalignment experienced by night or rotating shift workers while maintaining basic circadian oscillation patterns. The sleep deprivation models were classified as having a moderate risk of bias, reflecting challenges in distinguishing direct circadian effects from stress responses and metabolic changes associated with sleep loss. Constant light exposure models have high risk of bias scores due to their potential for causing multiple physiological disruptions beyond circadian misalignment, including melatonin suppression, behavioral changes, and metabolic alterations that may independently affect cardiovascular outcomes.

The methodological distinctions must be considered when interpreting the translational relevance of findings. Human shift workers typically experience partial and recurrent circadian disruptions, which are more accurately mimicked by light phase shift protocols than by constant light exposure or complete genetic disruption of the molecular clock. Genetic models such as *Bmal1*, *Clock*, and *Nr1d1/Nr1d2* KO induce permanent and systemic circadian disruption. While these models are valuable to understand the core clock mechanisms, they may not fully replicate the gradual and intermittent misalignment experienced by human shift workers. Environmental models such as light phase shift and sleep deprivation induce more transient circadian disruption, potentially allowing adaptive physiological responses that complicate comparisons with genetic models. Furthermore, the majority of studies have used male animals despite known sex differences in circadian regulation and cardiovascular physiology. Evidence indicates that females show higher high-frequency heart rate variability, indicating parasympathetic dominance, whereas males exhibit higher low-frequency heart rate variability, reflecting stronger sympathetic modulation [74]. At the molecular level, the core circadian gene *Bmal1* mediates sex-specific cardiac gene expression in mice, as cardiomyocyte-specific *Bmal1* KO reduces differentially expressed genes between sexes by approximately eight-fold [75, 76]. Estrogen appears to be protective against CVD in premenopausal women by modulating circadian clock gene expression. However, post-menopausal reductions in estrogen level and increases in testosterone level diminish this cardioprotective effect, elevating hypertension and coronary artery disease risk [74, 77]. Therefore, the predominant use of male animals in the included studies (65.6%) may limit the generalizability of findings to female populations, particularly relevant as many shift workers in healthcare and service industries are women.

In this systematic review we identified only studies on rodent models that fulfilled the predefined PECO-based inclusion criteria. While rodent models provide several advantages for investigating mechanistic research, limitations with respect to translational relevance need to be considered as their physiological and temporal characteristics differ from humans. Rodent models allow for high control of environmental conditions, targeted genetic manipulation, and access to cardiovascular tissues for molecular and functional assessment. In multiple studies, circadian disruption induced consistent alterations in key biomarkers such as EF, cardiomyocyte size, LV/BW, vasorelaxation, and markers of cardiac hypertrophy, fibrosis, and inflammation. These findings support the biological plausibility of circadian contributions to cardiovascular disease mechanisms. However, the key limitations of using rodent models should also be critically assessed, which may affect their translational relevance to human shift workers. The most fundamental limitation emerges from the inverse circadian biology between nocturnal rodents and diurnal humans [78], creating challenges particularly for environmental disruption models. This may explain why genetic KO models showed more robust and consistent effects than environmental interventions. Additionally, rodents exhibit different cardiovascular physiologies, including higher HDL cholesterol levels and resistance to spontaneous atherosclerosis [72], which likely explains the inconsistent lipid findings observed in the included studies. Rodent studies also capture disease development over compressed timelines (weeks to months), which may not reflect the chronic progression of cardiovascular conditions in humans. Furthermore, constant light protocols, which are frequently used in animal models, can introduce systemic stressors unrelated to circadian disruption [79]. Overall, this systematic review highlights the strengths of animal models in providing insight into circadian-related mechanisms of cardiovascular dysfunction, while also underscoring the need for careful interpretation, extrapolation, and validation of these findings in large animal models and human cohorts to confirm the translational applicability of the mechanisms [80].

## Conclusions

This systematic review and meta-analysis demonstrate that cardiac hypertrophy is the most robust finding in rodent models of circadian disruption, with high-certainty evidence for increased LV/BW and moderate-certainty evidence for cardiomyocyte enlargement. The evidence for other cardiovascular outcomes has remained predominantly low to very low certainty. The substantial methodological heterogeneity across in vivo studies significantly limits the confidence of the evidence. Thus, future in vivo research should prioritize standardizing experimental protocols to facilitate cross-study comparisons, incorporate sex-balanced designs given the predominant use of male animals, and distinguish between acute and chronic circadian disruption effects relevant to human shift workers experiencing intermittent rather than permanent disruption. While cardiac hypertrophy markers showed the most consistent effects in animal models, substantial epidemiological knowledge gaps remain, warranting further investigation of these biomarkers in shift workers.

## Supplementary Information

Additional file 1: Supplementary information. PRISMA 2020 checklist; database search strategies (MEDLINE/Embase/Web of Science); eligibility criteria for inclusion and exclusion of TIAB and full-text screening; risk-of-bias details; GRADE profiles; forest and funnel plots (Supplementary Figs. S1–S8); study characteristics and key findings (Supplementary Tables 1–10).

Additional file 2. ToxRTool template.

Additional file 3. Data extraction template.

Additional file 4. Meta-analysis script.

## Data Availability

All data generated or analyzed during this study are included in this published article and its supplementary information files.

## Abbreviations

ANS: Autonomic nervous system
AT1R: Angiotensin type 1 receptor
*Bmal1*: Brain and muscle Arnt-like protein 1
*Ccl2*: Chemokine ligand 2
*Ccr2*: Chemokine receptor type 2
CI: Confidence interval
*Clock*: Circadian locomotor output cycles kaput
*Clockmut*: *Clock* Mutant
CRP: C-reactive protein
CVD: Cardiovascular disease
EF: Ejection fraction
ET1: Endothelin-1
FS: Fractional shortening
GRADE: Grading of Recommendations Assessment, Development and Evaluation
GSH: Reduced glutathione
HDL: High-density lipoprotein
*Il1b*: Interleukin 1 beta
IL6: Interleukin 6
IVRT: Isovolumetric relaxation time
IVS: Interventricular septum thickness
KO: Knockout
LV/BW: Left ventricular mass to body weight ratio
LVID: Left ventricular internal diameter
LVPWd: Left ventricular posterior wall thickness at diastole
NOS3: Nitric oxide synthase 3
*Nr1d1*: Nuclear receptor subfamily 1 group D member 1
*Nr1d2*: Nuclear receptor subfamily 1 group D member 2
PAI1: Plasminogen activator inhibitor-1
PECO: Population, Exposure, Comparator, Outcome
PRISMA: Preferred Reporting Items for Systematic Reviews and Meta-Analyses
ROS: Reactive oxygen species
RR: Risk ratio
SCN: Suprachiasmatic nucleus
SD: Standard deviation
SMD: Standardized mean difference
TC: Total cholesterol
TG: Triglycerides
*Tnf*: Tumor necrosis factor
