# Supplementary material for: Circadian rhythm disruption in cardiovascular disease: a systematic review and meta-analysis of mechanistic evidence from animal models
Source: BMC Med. 2026 Jan 9;24:73. doi: 10.1186/s12916-025-04572-3 (PMC12882426; doi:10.1186/s12916-025-04572-3)
Supplement: Supplementary file 1 — Additional file 1: Supplementary information. PRISMA 2020 checklist; database search strategies (MEDLINE/Embase/Web of Science); eligibility criteria for inclusion and exclusion of TIAB and full-text screening; risk-of-bias details; GRADE profiles; forest and funnel plots (Supplementary Figs. S1–S8); study characteristics and key findings (Supplementary Tables 1–10). [file 12916_2025_4572_MOESM1_ESM.docx]

**Supplementary Information**

**Circadian Rhythm Disruption in Cardiovascular Disease: A Systematic Review of *In Vivo* Mechanistic Evidence**

Mrinal K. Das^a^, Evi De Ryck^b^, Ingrid L. Jorgensen^a^, Shan Zienolddiny-Narui^a^, Johanna Samulin Erdem^a1^

^a^National Institute of Occupational Health, Oslo, Norway; ^b^Department of Public Health and Primary Care, KU Leuven, Leuven, Belgium

^1^Corresponding author: Johanna Samulin Erdem; Pb 5330, 0304 Oslo, Norway; (+47)23195100; johanna.samulin-erdem@stami.no

**Supplementary figures**


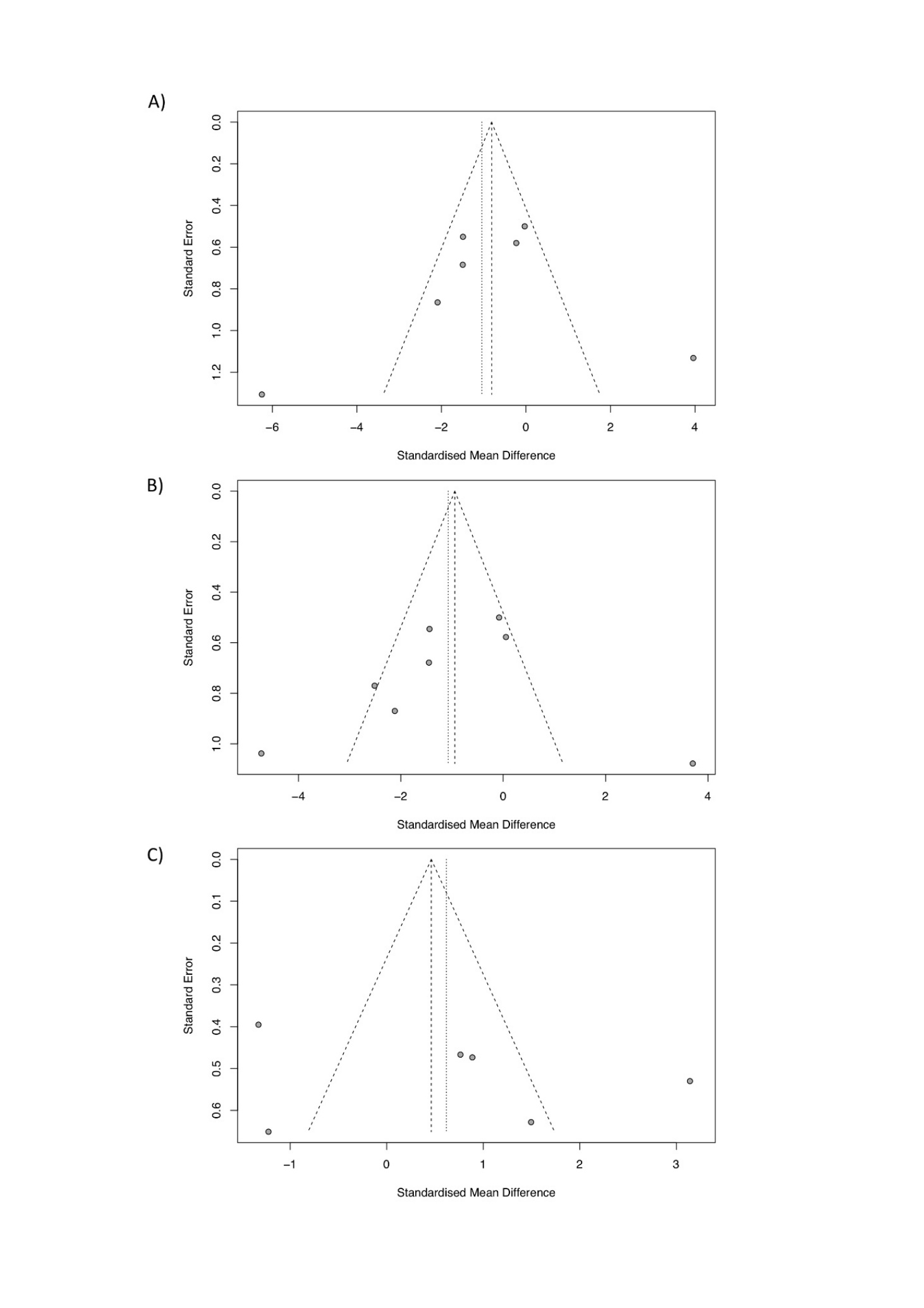


Figure S1. Funnel plots of A) EF, B) FS, and C) LVID


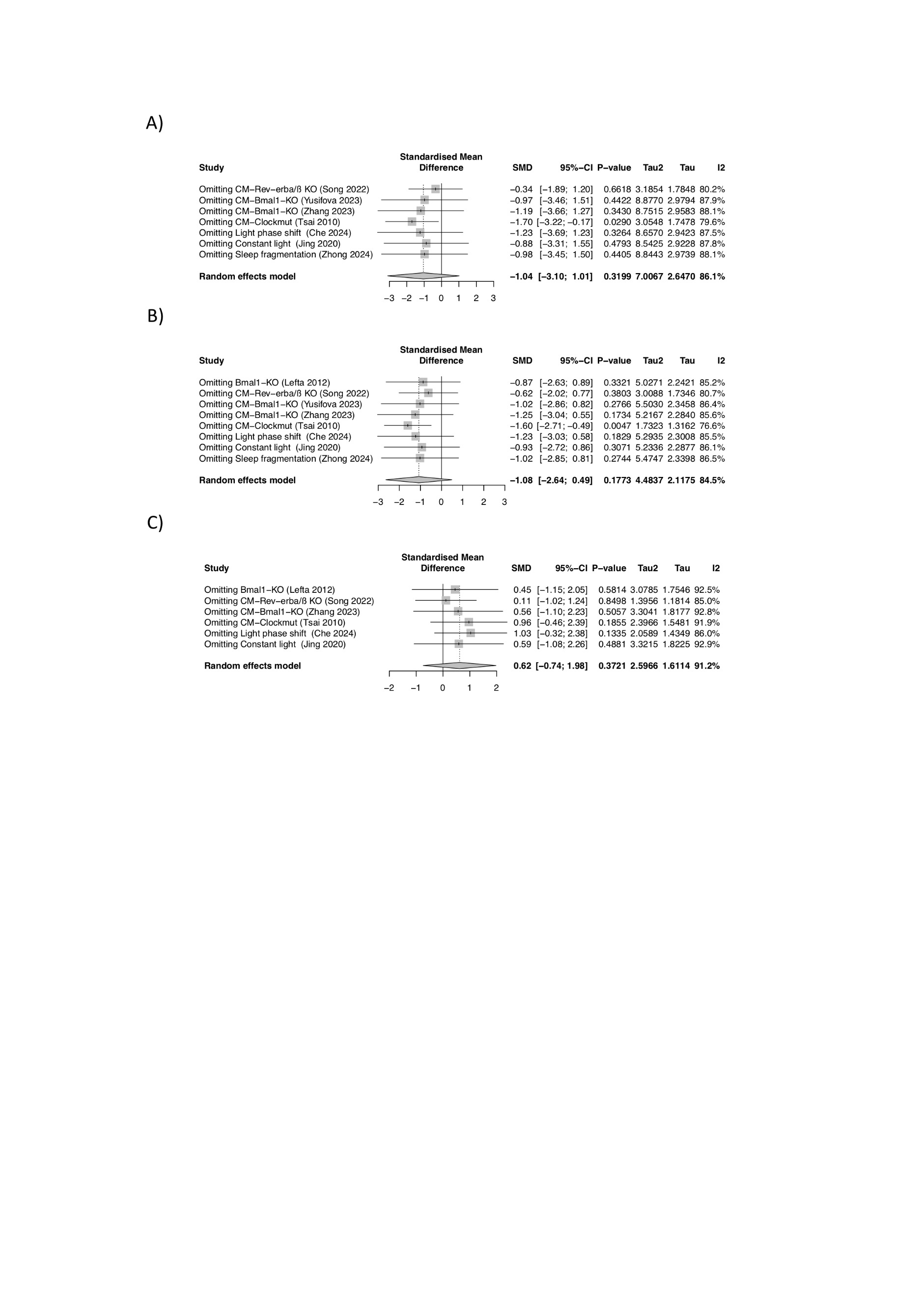


Figure S2. Leave-out-one sensitivity analysis illustrated by forest plots for A) EF, B) FS, and C) LVID.


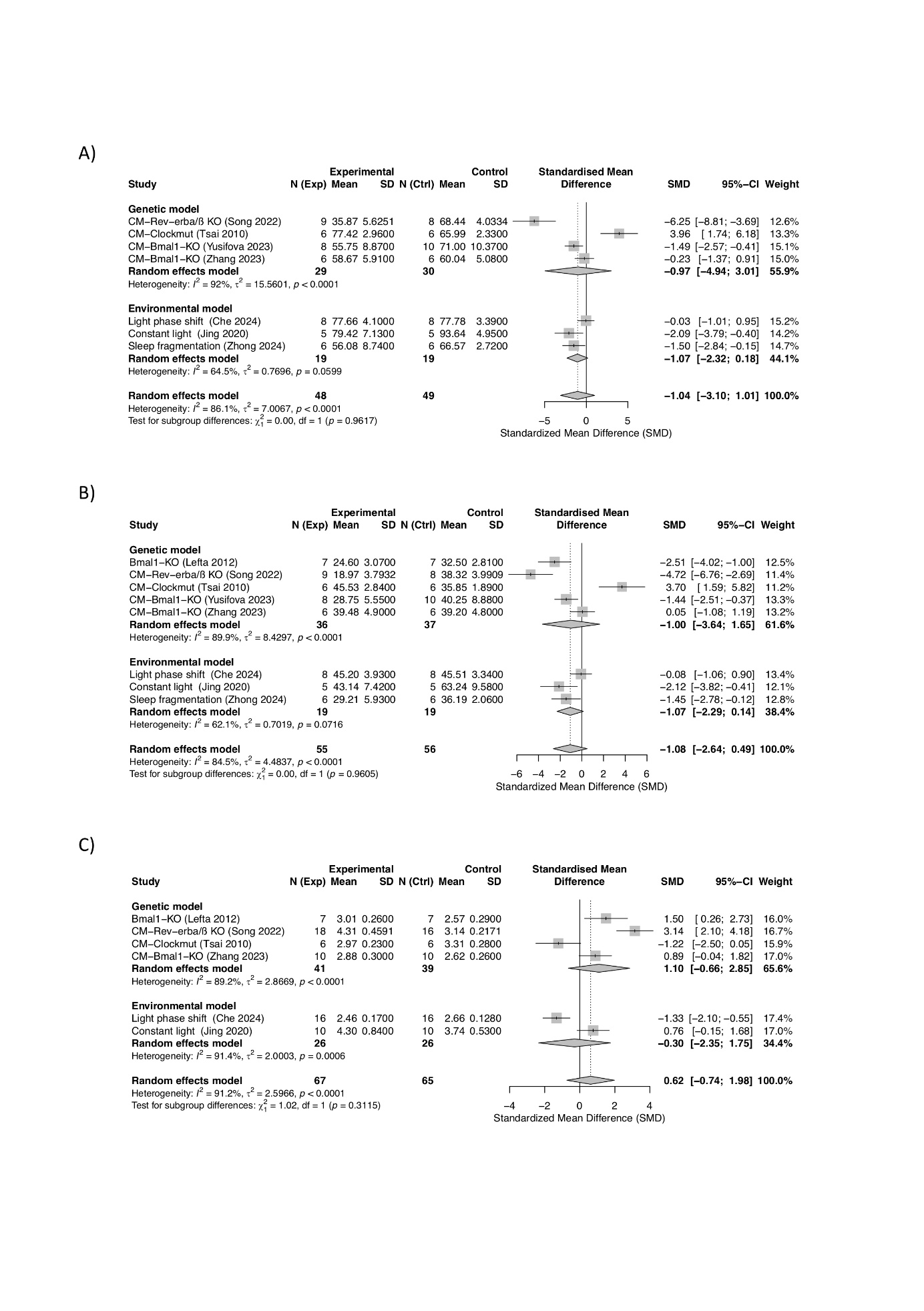


Figure S3. Subgroup analysis illustrated by forest plots for A) EF, B) FS, and C) LVID.


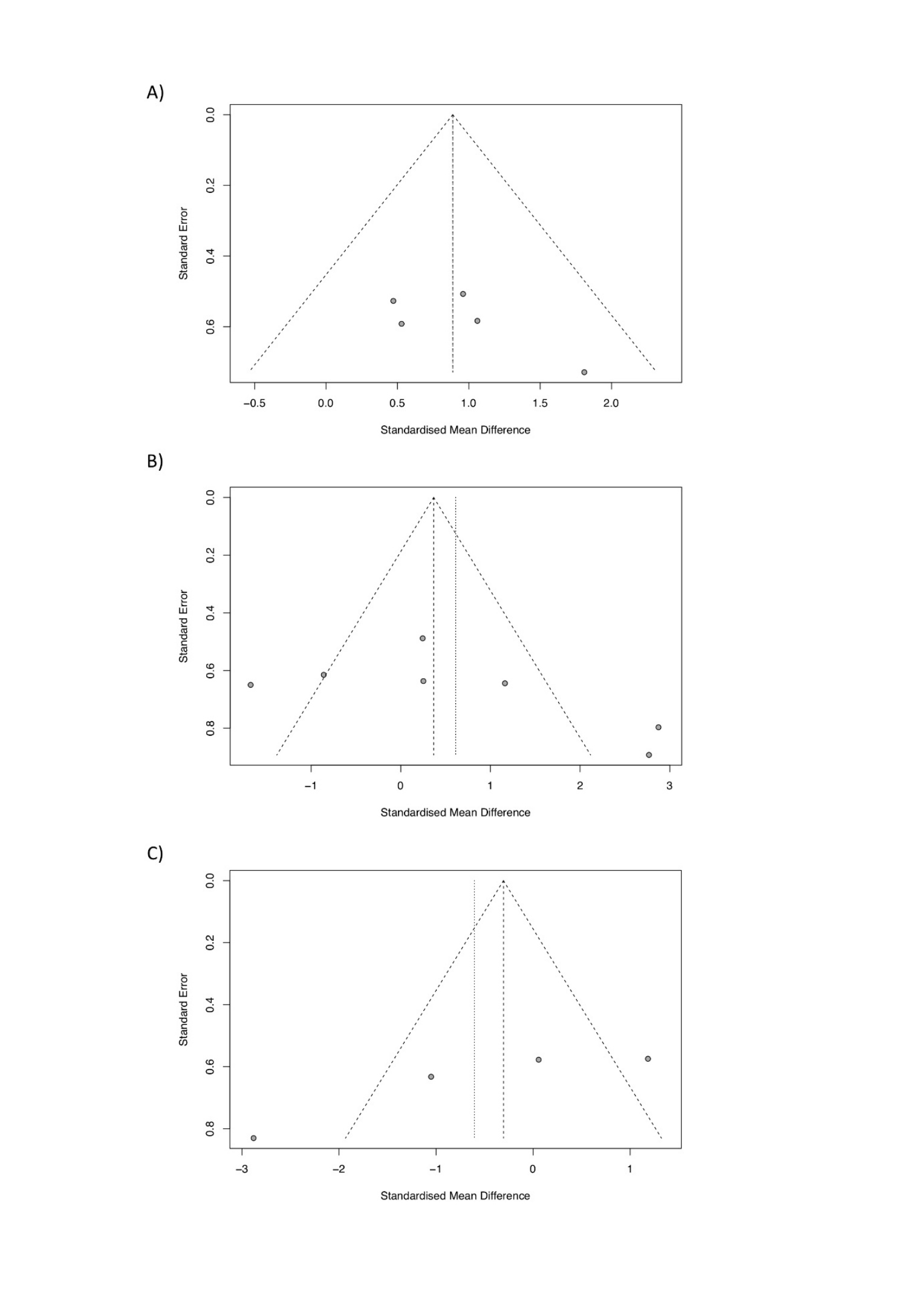


Figure S4. Funnel plots of A) LV/BW, B) LVPWd thickness and C) IVS thickness


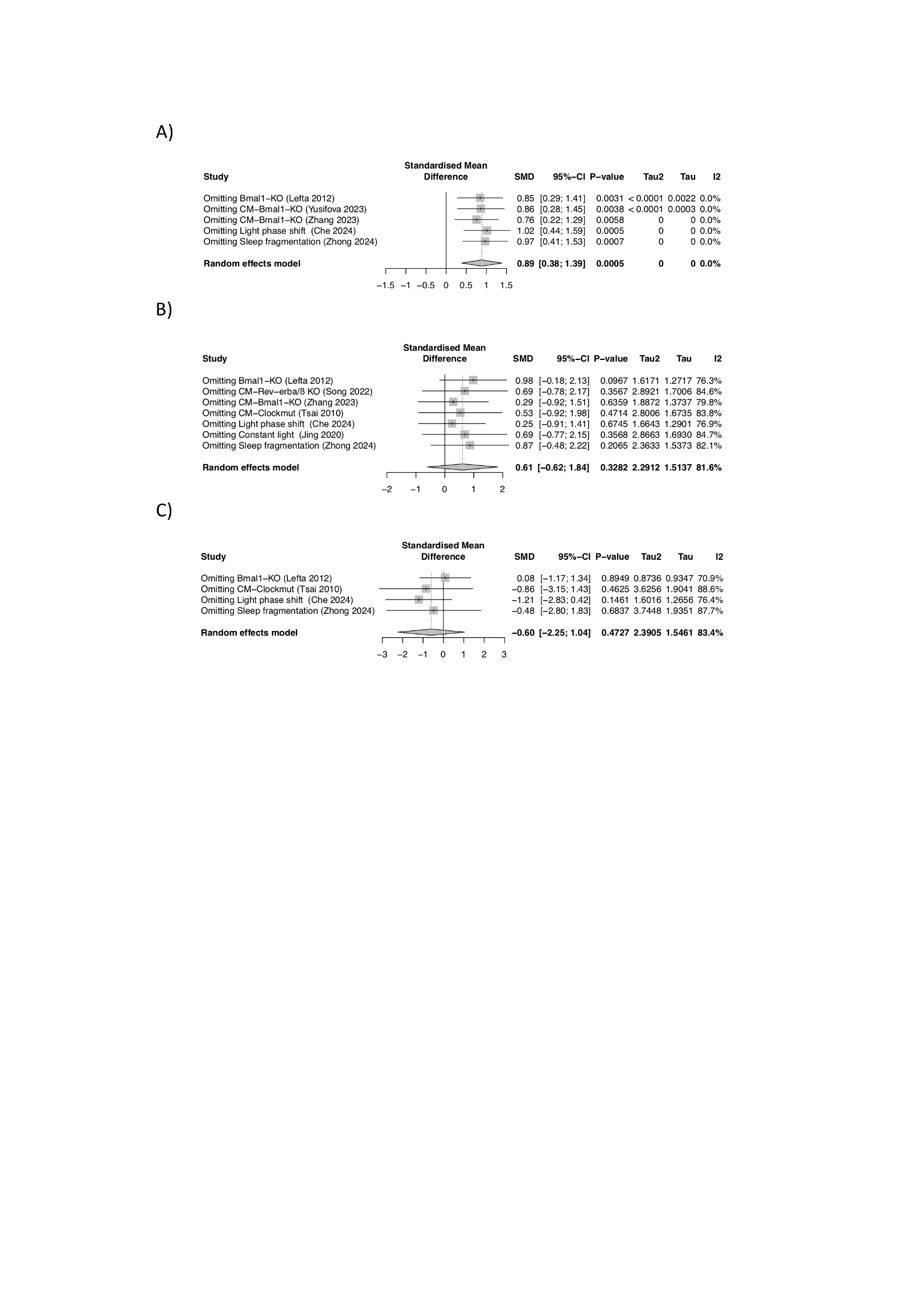


Figure S5. Leave-out-one sensitivity analysis illustrated by forest plots for A) LV/BW, B) LVPWd and C) IVS.


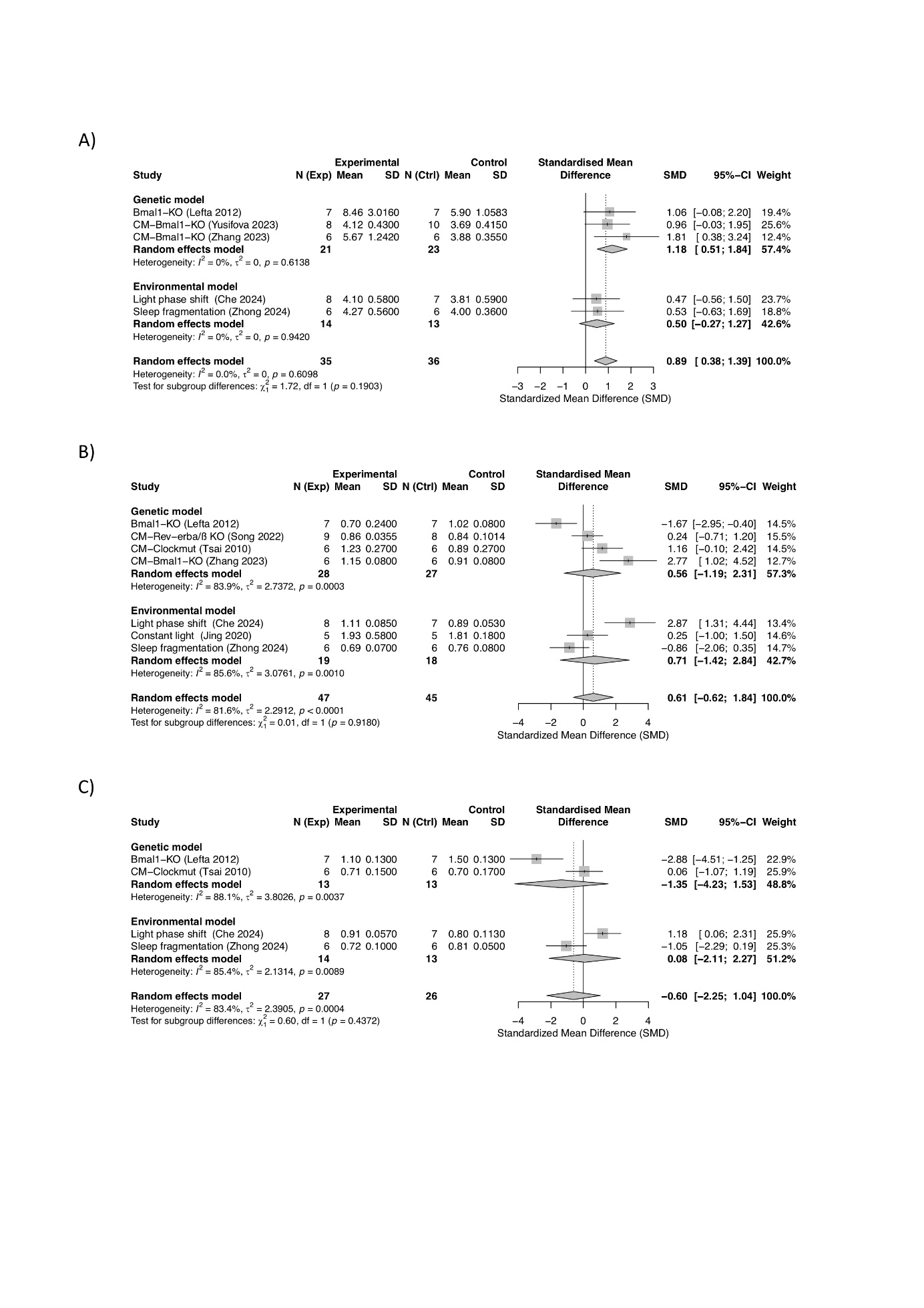


Figure S6. Subgroup analysis illustrated by forest plots for A) LV/BW, B) LVPWd and C) IVS.


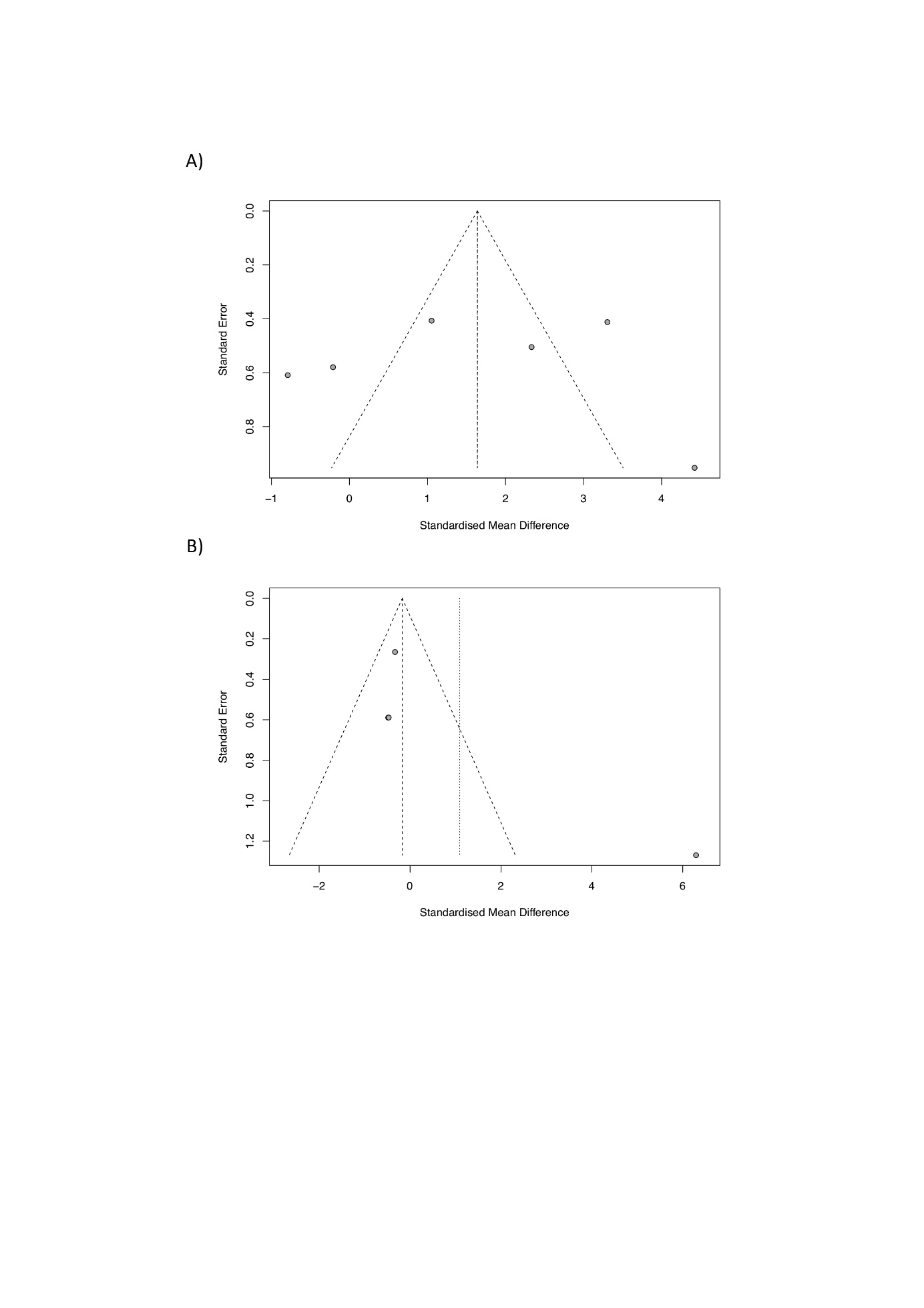


Figure S7. Funnel plots of A) TG and B) TC


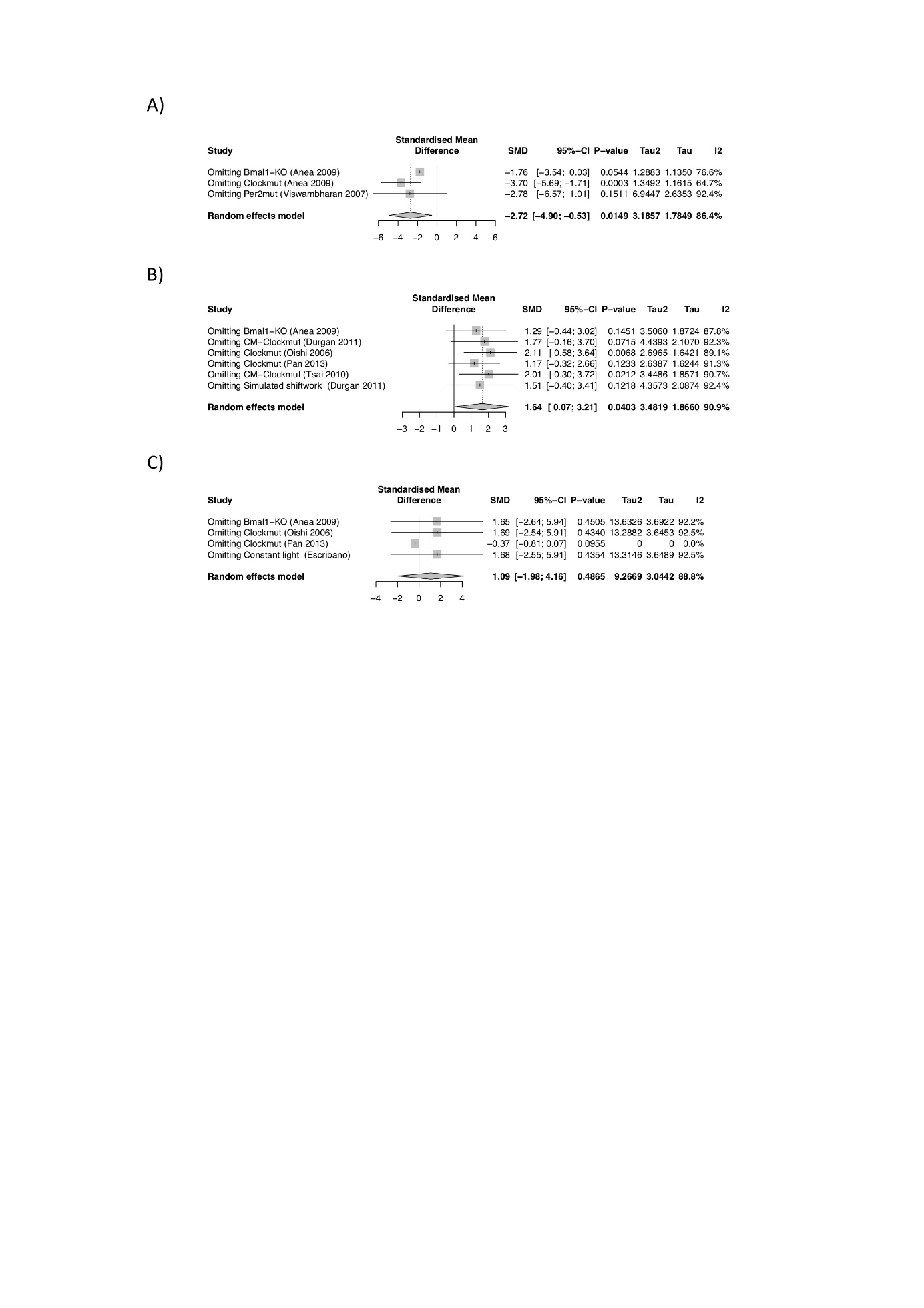
Figure S8. Leave-out-one sensitivity analysis illustrated by forest plots for A) Vasorelaxation, B) TG and C) TC.

**Supplementary tables**

Supplementary Table 1: PRISMA 2020 Checklist [1].

| **Section and Topic** | **Item #** | **Checklist item** | **Reported in section #** |
| --- | --- | --- | --- |
| **TITLE** | | |  |
| Title | 1 | Identify the report as a systematic review. | Title |
| **ABSTRACT** | | |  |
| Abstract | 2 | See the PRISMA 2020 for Abstracts checklist. | Abstract |
| **INTRODUCTION** | | |  |
| Rationale | 3 | Describe the rationale for the review in the context of existing knowledge. | Introduction |
| Objectives | 4 | Provide an explicit statement of the objective(s) or question(s) the review addresses. | Introduction |
| **METHODS** | | |  |
| Eligibility criteria | 5 | Specify the inclusion and exclusion criteria for the review and how studies were grouped for the syntheses. | Methods |
| Information sources | 6 | Specify all databases, registers, websites, organizations, reference lists and other sources searched or consulted to identify studies. Specify the date when each source was last searched or consulted. | Methods |
| Search strategy | 7 | Present the full search strategies for all databases, registers and websites, including any filters and limits used. | Tables S2-4 |
| Selection process | 8 | Specify the methods used to decide whether a study met the inclusion criteria of the review, including how many reviewers screened each record and each report retrieved, whether they worked independently, and if applicable, details of automation tools used in the process. | Methods |
| Data collection process | 9 | Specify the methods used to collect data from reports, including how many reviewers collected data from each report, whether they worked independently, any processes for obtaining or confirming data from study investigators, and if applicable, details of automation tools used in the process. | Methods |
| Data items | 10a | List and define all outcomes for which data were sought. Specify whether all results that were compatible with each outcome domain in each study were sought (e.g. for all measures, time points, analyses), and if not, the methods used to decide which results to collect. | Table S7-8 |
|  | 10b | List and define all other variables for which data were sought (e.g. participant and intervention characteristics, funding sources). Describe any assumptions made about any missing or unclear information. | Tables S5 and S7 |
| Study risk of bias assessment | 11 | Specify the methods used to assess risk of bias in the included studies, including details of the tool(s) used, how many reviewers assessed each study and whether they worked independently, and if applicable, details of automation tools used in the process. | Methods, Tables 2, S6 |
| Effect measures | 12 | Specify for each outcome the effect measure(s) (e.g. risk ratio, mean difference) used in the synthesis or presentation of results. | Methods |
| Synthesis methods | 13a | Describe the processes used to decide which studies were eligible for each synthesis (e.g. tabulating the study intervention characteristics and comparing against the planned groups for each synthesis (item #5)). | Methods |
|  | 13b | Describe any methods required to prepare the data for presentation or synthesis, such as handling of missing summary statistics, or data conversions. | Methods |
|  | 13c | Describe any methods used to tabulate or visually display results of individual studies and syntheses. | Methods |
|  | 13d | Describe any methods used to synthesize results and provide a rationale for the choice(s). If meta-analysis was performed, describe the model(s), method(s) to identify the presence and extent of statistical heterogeneity, and software package(s) used. | Methods |
|  | 13e | Describe any methods used to explore possible causes of heterogeneity among study results (e.g. subgroup analysis, meta-regression). | Methods, Fig. S3, S6-9 |
|  | 13f | Describe any sensitivity analyses conducted to assess robustness of the synthesized results. | Methods, Fig. S 4,5,6 |
| Reporting bias assessment | 14 | Describe any methods used to assess risk of bias due to missing results in a synthesis (arising from reporting biases). | Methods, Fig S 1,2,3 |
| Certainty assessment | 15 | Describe any methods used to assess certainty (or confidence) in the body of evidence for an outcome. | Methods, Tables 2, S6 |
| **RESULTS** | | |  |
| Study selection | 16a | Describe the results of the search and selection process, from the number of records identified in the search to the number of studies included in the review, ideally using a flow diagram. | Results, Fig. 1 |
|  | 16b | Cite studies that might appear to meet the inclusion criteria, but which were excluded, and explain why they were excluded. | Results |
| Study characteristics | 17 | Cite each included study and present its characteristics. | Table 1, Table S7 |
| Risk of bias in studies | 18 | Present assessments of risk of bias for each included study. | Results; Tables S6 |
| Results of individual studies | 19 | For all outcomes, present, for each study: (a) summary statistics for each group (where appropriate) and (b) an effect estimates and its precision (e.g. confidence/credible interval), ideally using structured tables or plots. | Results |
| Results of syntheses | 20a | For each synthesis, briefly summarize the characteristics and risk of bias among contributing studies. | Results - Synthesis |
|  | 20b | Present results of all statistical syntheses conducted. If meta-analysis was done, present for each the summary estimate and its precision (e.g. confidence/credible interval) and measures of statistical heterogeneity. If comparing groups, describe the direction of the effect. | Results - Synthesis, Fig. 2-4, Fig. S1-9 |
|  | 20c | Present results of all investigations of possible causes of heterogeneity among study results. | Results – Synthesis, Fig. 2-4, Fig. S7-9 |
|  | 20d | Present results of all sensitivity analyses conducted to assess the robustness of the synthesized results. | Results – Synthesis, Fig. S4-6 |
| Reporting biases | 21 | Present assessments of risk of bias due to missing results (arising from reporting biases) for each synthesis assessed. | Results – Table S6 |
| Certainty of evidence | 22 | Present assessments of certainty (or confidence) in the body of evidence for each outcome assessed. | Results, Table 2, Table S9 |
| **DISCUSSION** | | |  |
| Discussion | 23a | Provide a general interpretation of the results in the context of other evidence. | Discussion – Summary of findings |
|  | 23b | Discuss any limitations of the evidence included in the review. | Discussion – Strength and limitations |
|  | 23c | Discuss any limitations of the review processes used. | Discussion – Strength and limitations |
|  | 23d | Discuss implications of the results for practice, policy, and future research. | Discussion – Conclusions and future directions |
| **OTHER INFORMATION** | | |  |
| Registration and protocol | 24a | Provide registration information for the review, including register name and registration number, or state that the review was not registered. | Methods |
|  | 24b | Indicate where the review protocol can be accessed, or state that a protocol was not prepared. | Not prepared |
|  | 24c | Describe and explain any amendments to information provided at registration or in the protocol. | Methods |
| Support | 25 | Describe sources of financial or non-financial support for the review, and the role of the funders or sponsors in the review. | Funding sources |
| Competing interests | 26 | Declare any competing interests of review authors. | Declaration of interests |
| Availability of data, code and other materials | 27 | Report which of the following are publicly available and where they can be found: A) template data collection forms; B) data extracted from included studies, data used for all analyses; C) analytic code; D) any other materials used in the review. | Supplementary data |

*From:*  Page MJ, McKenzie JE, Bossuyt PM, Boutron I, Hoffmann TC, Mulrow CD, et al. The PRISMA 2020 statement: an updated guideline for reporting systematic reviews. BMJ 2021;372:n71. doi: 10.1136/bmj.n71

Supplementary Table 2: Search strategy in Medline.

| 1. | cardiovascular diseases/ or exp heart diseases/ or exp vascular diseases/ |
| --- | --- |
| 2. | (arterial or "arterial ischaemi*" or "arterial ischemi*" or "ischaemic event*" or "ischemic event*" or "arterial occlusion*" or "occlusive event*" or "arterial thrombosis" or atherothrombosis or athero-thrombosis or "athero thrombosis" or athero* or vascular or "cardiovascular diseas*" or "cardio vascular diseas*" or "cardiovascular ischaemi*" or "cardiovascular ischemi*" or "cardio vascular ischaemi*" or "cardio vascular ischemi*" or stroke or "ischaemic stroke" or "ischemic stroke" or "brain infarction" or "brain ischaemi*" or "brain ischemi*" or "transient ischaemic attack" or "transient ischemic attack" or heart or "heart attack" or "heart infarction" or "myocardial infarction" or "myo-cardial infarction" or "myo cardial infarction" or myocardi* or myo-cardi* or "myo cardi*" or "coronary artery occlusion" or "coronary artery ischaemi*" or "coronary artery ischemi*" or "coronary heart diseas*" or "coronary artery" or atherosclerosis or athero-sclerosis or "athero sclerosis" or arrhythmia* or "ardiac inotrop*" or "cardiac lusitrop*" or "atrial fibrillation*" or "cardiomyocyte injur*" or "cardiomyocyte death*" or hypertroph* or cardiomyopath* or myocardiopath* or "cardiac injur*" or myocarditis or "valve stroma" or vasodilation* or "vascular disease*" or "vascular injur*" or "vascular dysfunction*" or hypertension* or "peripheral artery disease*" or thrombosis or microangiopath* or micro-angiopath* or "organ ischem*" or dyslipidemi* or "vascular fibrosis" or hyperlipidemia*).ti,ab. |
| 3. | 1 or 2 |
| 4. | exp Shift Work Schedule/ |
| 5. | chronobiology disorders/ or exp sleep disorders, circadian rhythm/ |
| 6. | biological clocks/ |
| 7. | ("shift work*" or shift-work* or shiftwork* or "night-shift work*" or "night shift work*" or "rotating shift*" or rotating-shift* or "night work*" or "night shift*" or nightshift* or three-shift* or "three shift*" or two-shift* or "two shift*" or graveyard-shift* or "graveyard shift*" or nightdut* or night-dut* or "night dut*" or "circadian misalignment*" or "circadian desynchronization*" or "circadian desynchron*" or "circadian disruption*" or "chronobiology disorder*" or "biological clock*" or "circadian rhythm").ti,ab. |
| 8. | 4 or 5 or 6 or 7 |
| 9. | 3 and 8 |
| 10. | limit 9 to English |
| 11. | limit 10 to (case reports or classical article or comparative study or "corrected and republished article" or duplicate publication or evaluation study or journal article or observational study or preprint or randomized controlled trial or technical report or validation study) |
| 12. | limit 10 to (meta analysis or "review" or "systematic review") |
| 13. | 11 not 12 |

Supplementary Table 3. Search strategy in Embase.

| 1. | cardiovascular disease/ or exp heart disease/ or exp vascular disease/ |
| --- | --- |
| 2. | (arterial or "arterial ischaemi*" or "arterial ischemi*" or "ischaemic event*" or "ischemic event*" or "arterial occlusion*" or "occlusive event*" or "arterial thrombosis" or atherothrombosis or athero-thrombosis or "athero thrombosis" or athero* or vascular or "cardiovascular diseas*" or "cardio vascular diseas*" or "cardiovascular ischaemi*" or "cardiovascular ischemi*" or "cardio vascular ischaemi*" or "cardio vascular ischemi*" or stroke or "ischaemic stroke" or "ischemic stroke" or "brain infarction" or "brain ischaemi*" or "brain ischemi*" or "transient ischaemic attack" or "transient ischemic attack" or heart or "heart attack" or "heart infarction" or "myocardial infarction" or "myo-cardial infarction" or "myo cardial infarction" or myocardi* or myo-cardi* or "myo cardi*" or "coronary artery occlusion" or "coronary artery ischaemi*" or "coronary artery ischemi*" or "coronary heart diseas*" or "coronary artery" or atherosclerosis or athero-sclerosis or "athero sclerosis" or arrhythmia* or "ardiac inotrop*" or "cardiac lusitrop*" or "atrial fibrillation*" or "cardiomyocyte injur*" or "cardiomyocyte death*" or hypertroph* or cardiomyopath* or myocardiopath* or "cardiac injur*" or myocarditis or "valve stroma" or vasodilation* or "vascular disease*" or "vascular injur*" or "vascular dysfunction*" or hypertension* or "peripheral artery disease*" or thrombosis or microangiopath* or micro-angiopath* or "organ ischem*" or dyslipidemi* or "vascular fibrosis" or hyperlipidemia*).ti,ab. |
| 3. | 1 or 2 |
| 4. | exp shift schedule/ |
| 5. | exp circadian rhythm sleep disorder/ |
| 6. | biological rhythm/ |
| 7. | ("shift work*" or shift-work* or shiftwork* or "night-shift work*" or "night shift work*" or "rotating shift*" or rotating-shift* or "night work*" or "night shift*" or nightshift* or three-shift* or "three shift*" or two-shift* or "two shift*" or graveyard-shift* or "graveyard shift*" or nightdut* or night-dut* or "night dut*" or "circadian misalignment*" or "circadian desynchronization*" or "circadian desynchron*" or "circadian disruption*" or "chronobiology disorder*" or "biological clock*" or "circadian rhythm").ti,ab. |
| 8. | 4 or 5 or 6 or 7 |
| 9. | 3 and 8 |
| 10. | limit 9 to English |
| 11. | limit 10 to Embase |
| 12. | limit 11 to (article or article in press or "preprint (unpublished, non-peer reviewed)") |
| 13. | limit 11 to "review" |

Supplementary Table 4: Search strategy in Web of Science.

| 1. | (TS=((arterial or "arterial ischaemi*" or "arterial ischemi*" or "ischaemic event*" or "ischemic event*" or "arterial occlusion*" or "occlusive event*" or "arterial thrombosis" or atherothrombosis or athero-thrombosis or "athero thrombosis" or athero* or vascular or "cardiovascular diseas*" or "cardio vascular diseas*" or "cardiovascular ischaemi*" or "cardiovascular ischemi*" or "cardio vascular ischaemi*" or "cardio vascular ischemi*" or stroke or "ischaemic stroke" or "ischemic stroke" or "brain infarction" or "brain ischaemi*" or "brain ischemi*" or "transient ischaemic attack" or "transient ischemic attack" or heart or "heart attack" or "heart infarction" or "myocardial infarction" or "myo-cardial infarction" or "myo cardial infarction" or myocardi* or myo-cardi* or "myo cardi*" or "coronary artery occlusion" or "coronary artery ischaemi*" or "coronary artery ischemi*" or "coronary heart diseas*" or "coronary artery" or atherosclerosis or athero-sclerosis or "athero sclerosis" or arrhythmia* or "ardiac inotrop*" or "cardiac lusitrop*" or "atrial fibrillation*" or "cardiomyocyte injur*" or "cardiomyocyte death*" or hypertroph* or cardiomyopath* or myocardiopath* or "cardiac injur*" or myocarditis or "valve stroma" or vasodilation* or "vascular disease*" or "vascular injur*" or "vascular dysfunction*" or hypertension* or "peripheral artery disease*" or thrombosis or microangiopath* or micro-angiopath* or "organ ischem*" or dyslipidemi* or "vascular fibrosis" or hyperlipidemia*))) AND TS=( ("shift work*" or shift-work* or shiftwork* or "night-shift work*" or "night shift work*" or "rotating shift*" or rotating-shift* or "night work*" or "night shift*" or nightshift* or three-shift* or "three shift*" or two-shift* or "two shift*" or graveyard-shift* or "graveyard shift*" or nightdut* or night-dut* or "night dut*" or "circadian misalignment*" or "circadian desynchronization*" or "circadian desynchron*" or "circadian disruption*" or "chronobiology disorder*" or "biological clock*" or "circadian rhythm")) |
| --- | --- |

Supplementary Table 5: Detailed eligibility criteria for TIAB (title and abstract) and full text screening. This table presents the detailed inclusion and exclusion criteria organized by 12 pre-specified Key Characteristics (KCs) of cardiovascular effects [2]. KCs represent distinct cardiovascular disease phenotypes and mechanisms rather than specific biomarkers, allowing inclusion of studies using diverse measurement approaches.

| **Criterion** | | **Inclusion criteria** | | **Exclusion criteria** |
| --- | --- | --- | --- | --- |
| Population (P) | | Adult mammalian models (rodents, pigs, dogs, rabbits, non-human primates); sexually mature animals (>8 weeks for rodents) | | Non-mammalian models, neonatal or juvenile animals |
| Exposure (E) | | Genetic modifications of clock genes (e.g. *Bmal1*, *Clock*, *Nr1d1/Nr1d2*, *Per*, *Cry* KO or mutations) or environmental interventions (light phase shifts, constant light exposure, sleep deprivation, sleep fragmentation) | | Studies on circadian disruption in diseased models |
| Comparator (C) | | Appropriate controls: wild-type littermate controls for genetic models or animals maintained under standard 12:12 hour light-dark cycles for environmental models | | No control group, inappropriate control conditions |
| Outcomes (O): KCs of cardiovascular effects | | | | |
| Domain 1: Cardiac structural phenotypes | | | | |
| KC1: Impairs regulation of cardiac excitability | Cardiac arrhythmia, abnormal action potential, cardiac conduction abnormalities, QT interval prolongation, ion channel dysfunction, hypoxia-induced arrhythmia | | Studies without relevant cardiac structural and functional phenotypes | |
| KC2: Impairs cardiac contractility and relaxation | Systolic dysfunction, diastolic dysfunction, impaired cardiac contractility, impaired cardiac relaxation, reduced cardiac output, atrial fibrillation, cardiomyocyte mechanical dysfunction | |  |  |
| KC3: Induces cardiomyocyte injury and death | Cardiac hypertrophy, pathological cardiac remodeling, cardiomyopathy, cardiac injury, myocarditis, cardiomyocyte death, cardiac fibrosis, hyalinization, vacuolation | |  |  |
| KC4: Induces proliferation of valve stroma | Valvular disease, valve stroma proliferation, valve interstitial cell proliferation | |  |  |
| Domain 2: Vascular phenotypes | | | | |
| KC5: Impacts endothelial and vascular function | Hypertension, hypotension, abnormal vascular reactivity, impaired vasodilation, abnormal vasoconstriction, endothelial dysfunction, peripheral artery disease, altered vascular permeability, impaired flow-mediated dilation | | Studies without relevant vascular and metabolic phenotypes | |
| KC6: Alters hemostasis | Abnormal hemostasis, increased bleeding tendency, thrombosis, microangiopathy, organ ischemia, abnormal platelet function, coagulopathy | |  |  |
| KC7: Causes dyslipidemia | Dyslipidemia, hyperlipidemia, abnormal lipid metabolism, atherosclerosis, vascular fibrosis, abnormal fat accumulation | |  |  |
| Domain 3: Mechanistic cardiovascular phenotypes | | | | |
| KC8: Impairs mitochondrial function | Mitochondrial dysfunction, impaired mitochondrial respiration, abnormal energy metabolism, defective electron transport chain function, altered mitochondrial antioxidant capacity | | Studies without relevant mechanistic cardiovascular phenotypes | |
| KC9: Modifies autonomic nervous system activity | Autonomic dysfunction, altered sympathetic tone, altered parasympathetic tone, abnormal heart rate variability, impaired baroreceptor sensitivity, altered autonomic cardiovascular regulation | |  |  |
| KC10: Induces oxidative stress | Oxidative stress, increased reactive oxygen species production, lipid peroxidation, protein oxidation, impaired antioxidant capacity, oxidative damage | |  |  |
| KC11: Causes inflammation | Cardiovascular inflammation, elevated inflammatory markers, increased cytokine production, endothelial activation, leukocyte infiltration, inflammatory cardiovascular disease | |  |  |
| KC12: Alters hormone signaling | Thyroid dysfunction (hyperthyroidism, hypothyroidism), abnormal thyroid hormone signaling, endocrine-mediated cardiovascular dysfunction | |  |  |
| OTHER ELIGIBILITY CRITERIA | | | | |
| Study design | Original research articles reporting experimental data from animal models | | Reviews, systematic reviews, meta-analyses, commentaries, editorials, book chapters, conference abstracts only | |
| Methodological reporting | Sufficient description of animal models, circadian disruption protocol, cardiovascular outcome assessment | | Incomplete method description preventing reliable data extraction | |
| Language | English language publications | | Non-English language publications | |
| Publication status | Published full-text articles and preprints with complete methods and results | | Conference abstracts only, unavailable full text after author contact | |

Note: Studies were eligible if they reported at least one cardiovascular phenotype within any of the 12 KCs. KCs represent distinct cardiovascular disease phenotypes. Studies using any validated method to assess these phenotypes were eligible for inclusion.

Supplementary Table 6: Additional data obtained through personal communication

| **Study ID** | **Contact Person** | **Valid Contact Address** | **Information Requested** | **Response Received (Y/N)** | **Information Obtained** | **Impact on Review** | **Date of Initial Contact** | **Date of Response** |
| --- | --- | --- | --- | --- | --- | --- | --- | --- |
| Anea 2010 | Rudic, Daniel | No | Additional details on animal age | No | No | Study excluded since age ≥8 weeks is an inclusion criteria | 24/08/2024 | N/A |
| Curtis 2007 | FitzGerald, Garret | Yes | Additional details on animal age | Yes | Yes | Study excluded since age ≥8 weeks is an inclusion criteria | 24/08/2024 | 29/08/2024 |
| Mia 2020 | Young, Martin E. | Yes | Additional details on animal age | Yes | Yes | Study included | 24/08/2024 | 28/08/2024 |
| Somanath 2011 | Somanath, Payaningal R. | No | Additional details on animal age | No | No | Study excluded since age ≥8 weeks is an inclusion criteria | 24/08/2024 | N/A |
| Tsai 2010 | Young, Martin E. | Yes | Additional details on animal age | Yes | Yes | Study included | 24/08/2024 | 28/08/2024 |
| Song 2022 | Sun, Zheng | Yes | Numerical data of EF, FS, LVID and LVPWd | Yes | Yes | Study included frommeta-analysis | 20/03/2025 | 20/03/2025 |
| Yusifova 2023 | Bruns, Danielle R | Yes | Numerical values of LV/BW for meta-analysis | Yes | Yes | Study included from meta-analysis | 20/03/2025 | 22/03/2025 |
| Oishi 2006 | Oishi, Katsutaka | Yes | Numerical values of TC(mg/dl) and TG (mg/dl) for meta-analysis | Yes | Yes | Study included from meta-analysis | 20/03/2025 | 20/03/2025 |
| Ferrell 2015 | Chiang, John Y L | Yes | Numerical values of TC(mg/dl) and TG (mg/dl) for meta-analysis | No | No | Study excluded from meta-analysis | 20/03/2025 | N/A |
| Viswabharan 2007 | Yang, Zhihong | Yes | Numerical values of TC(mg/dl) and TG (mg/dl) for meta-analysis | No | No | Study excluded from meta-analysis | 20/03/2025 | N/A |
| Li 2020 | Sun, Ning | XX | Numerical values of LVID (mm), IVS(mm) and EF(%) for meta-analysis | No | No | Study excluded from meta-analysis | 20/03/2025 | N/A |

Note: This table documents all attempts to contact study authors for additional information related to our systematic review. For each study where information was needed, we sent email requests to the corresponding authors using the most recent contact information available from published articles or institutional websites. A standardized email template was used for all initial contacts, with study-specific details included as needed. Follow-up emails were sent after two weeks if no response was received to the initial contact.

Supplementary Table 7: Risk of bias was averaged across four domains: population bias, intervention bias, analytical bias, and bias in design and reporting (assessed by ToxRTool). Each domain was scored as low (L = 1), moderate (M = 2), and high (H = 3). Population bias was based on sample size (high: n ≤ 5, moderate: 5 < n ≤ 10, low: n > 10). Intervention bias was categorized as low for tissue-specific genetic models and light phase shift, moderate for global genetic models and sleep deprivation, and high for constant light exposure or sleep fragmentation. Analytical method bias was low for physical measures (e.g., EF, FS), moderate for protein biomarkers (e.g., western blot, ELISA), and high for mRNA-based markers (e.g., qPCR). Reporting bias followed standard classification by the ToxRTool as category 1 (low), category 2 (moderate), and category 3 (high).

| **Study ID** | **Design and**  **Reporting** | **Population** | **Intervention** | **Analytical** | **Overall Bias Score** |
| --- | --- | --- | --- | --- | --- |
| Anea 2009 | L | M | M | L | **L** |
| Anea 2013 | M | H | M | M | **M** |
| Che 2024 | L | M | L | L | **L** |
| Cheng 2021 | L | L | M | M | **L** |
| Duan 2022 | L | M | L | L | **L** |
| Durgan 2006 | M | L | L | L | **L** |
| Durgan 2011 | L | H | L | M | **M** |
| Escribano 2014 | L | M | H | L | **M** |
| Ferrell 2015 | L | H | M | M | **M** |
| Gu 2024 | L | M | L | L | **M** |
| Hemmeryckx 2011 | L | M | M | L | **L** |
| Hou 2025 | L | M | M | M | **M** |
| Jing 2020 | L | H | H | L | **M** |
| Kadomatsu 2013 | L | H | M | M | **M** |
| Lefta 2012 | L | M | M | M | **M** |
| Li 2020 | L | L | M | L | **L** |
| Mia 2020 | M | M | L | L | **L** |
| Oishi 2006 | L | M | M | L | **L** |
| Pan 2013 | L | M | M | L | **L** |
| Periasamy 2015 | L | M | M | M | **M** |
| Sato 2023 | L | H | L | M | **M** |
| Schroder 2015 | L | H | L | L | **L** |
| Sutovska 2021 | L | M | H | M | **M** |
| Tsai 2010 | L | M | L | L | **L** |
| Viswambharan 2007 | L | M | M | M | **M** |
| Yusifova 2023 | L | H | L | L | **L** |
| Shi 2022 | L | M | L | L | **L** |
| Song 2022 | L | M | L | M | **L** |
| Wang 2023 | L | L | L | L | **L** |
| Zhong 2024 | L | M | H | L | **M** |
| Zhang 2023 | L | H | L | L | **L** |
| Zlobina 2021 | L | L | L | L | **L** |

Abbreviations: L: Low; M: Moderate; H: High

Supplementary Table 8: Study descriptives

| **Study ID** | **Species** | **Animal strain** | **Sex (M, F, NS)** | **Age** | **Body weight (g)** | **Population number** | **Type of intervention** | **Experiment design** | **Experimental duration/exposure time** | **Controls** |
| --- | --- | --- | --- | --- | --- | --- | --- | --- | --- | --- |
| Anea 2009 | Mice | C57BL/6J | M, F | 7-10 and 25-30 weeks | 14.9-21.1 | 5-14 per group | *Bmal1* KO, *Clockmut* | *Bmal1* KO mice kept at 12:12 LD conditions and Clockmut mice kept at 12:12 LD or DD conditions. | 5 weeks | WT mice (12:12 LD) |
| Anea 2013 | Mice | NS | NS | 6-16 weeks | 22-32 | 3-7 per group | *Bmal1* KO | NA | NA | WT mice |
| Che 2024 | Mice | C57BL/6 | M | 8-10 weeks | NS | 5-8 per group | Light phase shift/ circadian disruption | Mice were subjected to 8h advances of light periods once every 4 days for up to 3 months. | 1-3 months | Normal light (12:12 LD) |
| Cheng 2021 | Mice | ICR | M | 8 weeks | NS | 20 per group | *Clock* knockdown | *Clock* expression was downregulated by Clock shRNA plasmid transfection. | 2 days | Saline |
| Duan 2022 | Rat | Sprague-Dawley | M | 8-12 weeks | 200-220 | 5-7 per group | Light phase shift/ circadian disruption | 12:12 LD (light from 8 a.m.-8 p.m. [zeitgeber time (ZT) 0-ZT12], and dark from 8 p.m.-8 a.m. (ZT12-ZT24)). For circadian disruption, light-onset time was advanced 6h every two days for 42 days. | 42 days | Normal light (12:12 LD) |
| Durgan 2006 | Mice | NS | NS | 8 weeks | NS | 10-17 per group | Cardiomyocyte *Clockmut* | The mice had a cardiomyocyte selective expression of a dominant negative *Clockmut* protein. The expression of a truncated clock gene, lacking a functional transactivation domain, was driven by the myosin heavy chain promoter, which targeted expression in cardiomyocytes specifically. | NA | WT mice |
| Durgan 2011 | Mice | FVB/N, C57BL/6J | M | 8 weeks, 2 or 18 months | NS | 3-7 per group | Cardiomyocyte *Clockmut*; simulated shift work | *Clockmut* mice used in a simulated shift work study where the animals were introduced to a bi-weekly 12h phase shift in the light/dark cycle for a total of 16 weeks | 16 weeks | WT mice, normal LD cycle |
| Escribano 2014 | Rat | Wistar | M | 3 months | 250-300 | 6 per group | Light/dark exposure | Rats were exposed to permanent likght (24h L) or complete darkness (22h D) | 2 weeks | Normal light (12:12 LD) |
| Ferrell 2015 | Mice | B6xC57 | F | 2-4 months | NS | 5-6 per group | Sleep disruption | Sleep-disruption were performed using gentle stimulation (brush and air) for 6 hours/day for 5 days during the middle of the light phase (ZT 2 – ZT 8). | 5 days | No intervention |
| Gu 2024 | Rat | Sprague-Dawley | M | 12 weeks | NS | 6 per group | Light phase shift/ circadian disruption | Circadian disruption by alternation of the light-dark cycle every third day | 12 weeks | Normal light (12:12 LD) |
| Hemmeryckx 2011 | Mice | C57BL/6J | M | 10 or 30 weeks | WT:20.5-33, KO: 17-25 | 4-8 per group | *Bmal1* KO | NA | NA | WT mice |
| Hou 2025 | Mice | C57BL/6 × 129SvEv | M | 10-20 weeks | NS | 5-10 per group | Cardiomyocyte-specific *Bmal1* KO | *Bmal1* flox/flox mice were crossed with the UBC-Cre-ERT2+/− mice. Tamoxifen (2 mg/day/mouse) was administred byintraperitoneal injection, for 5 consecutive days, in the *Bmal1*flox/flox//UBC-CreERT+/− mice. | NA | WT mice |
| Jing 2020 | Rat | Sprague-Dawley | M | NS | 220-270 | 3-5 per group | Light exposure | Constant light | 4 weeks | Normal light (12:12 LD) |
| Kadomatsu 2013 | Mice | C57BL/6J | M | 2-5 months | NS | 3-6 per group | *Cry1/2* KO | Consant darkness for 36 hours | 36h | WT mice |
| Lefta 2012 | Mice | C57BL/6J | NS | 8-36 weeks | NS | 4-8 per group | *Bmal1* KO | NA | NA | WT mice |
| Li 2020 | Mice | NS | NS | 20-32 weeks | 20-35 | 12 per group | Cardiomycyte *Bmal1mut*, *Bmal1* KO | Bmal1mut using CRISPR | NA | WT mice |
| Mia 2020 | Mice | C57BL/6J | M | >8 weeks | NS | 6-8 per group | Cardiomyocyte *Bmal1* KO | NA | NA | WT mice |
| Oishi 2006 | Mice | BALB/c and C57BL/6J backcrossed with Jcl:ICR mice | M | 8-9 weeks | NS | 6 per group | *Clockmut* mice | NA | NA | WT mice |
| Pan 2013 | Mice | C57BL/6J | NS | 2-3 months | NS | 6-12 per group | *Clockmut* mice | NA | 2 months | WT mice |
| Periasamy 2015 | Mice | C57BL/6J | M | 7-8 weeks | 25-30 | 6-7 per group | Sleep disruption | A modified multiple platform method was used (acrylic tank (40 x 30 cm) with 12 columns (platforms, 5 x 3 cm) filled with 1 cm water). | 24-72h | No intervention |
| Sato 2023 | Mice | C57BL/6J | M | 14 weeks (KO), 6-8 weeks (WT) | NS | 3 per group | Cardiac-specific *Bmal1* KO (*Myh6-Cre;Bmal1*flx/flx) | NA | NA | WT mice |
| Schroder 2015 | Mice | C57Bl/6 | M/F | 14–16 weeks | NS | 3-7 per group | Inducible *Bmal1* KO, DD exposure | 30 hours in D/D, the ventricular apex was collected every 4 hours from 3–4 animals in each group for a total of 8 time points. | 30h | WT mice |
| Shi 2022 | Mice | C57BL/6J | M/F | 8-12 weeks | NS | 8-12 per group | *Nr1d1* mice and platelet-specific *Nr1d1* KO mice | NA | NA | WT mice |
| Song 2022 | Mice | C57BL/6J | M/F | 2-8 months | NS | 3-13 per group | Cardiac-specific  *Nr1d1/Nr1d2* double KO | NA | NA | WT mice bearing *Nr1d1/Nr1d2*loxP alleles without Cre |
| Sutovska 2021 | Rat | Wistar | M | 4.5 months | 325±18 | 4-8 per group | Light exposure | Experimental artificial light at night (12 h light (150 lx)–12 h dim light (1−2 lx)) groups. | 2 and 5 weeks | Normal light (12:12 LD) |
| Tsai 2010 | Mice | FVB/N | M | >8 weeks | NS | 6 per group | Cardiomyocyte *Clockmut* | NA | 12 and 16 weeks | WT mice |
| Viswambharan 2007 | Mice | C57BL/6J | M | 3 months | NS | 5-9 per group | *Per2mut* mice | NA | NA | WT mice |
| Wang 2023 | Mice | C57BL/6 | M | 8 weeks | 27.5 | 12 per group | Light phase shift/ circadian disruption | The light cycle was delayed backwards by 4h every 3 days so that the model mice were in a continuously rolling light cycle. | 8 weeks | Normal light (12:12 LD) |
| Yusifova 2023 | Mice | C57BL/6 | M, F | 8 weeks | NS | 3-6 per group | Cardiomyocyte *Bmal1* KO | Mice homozygous for the Bmal1-loxP- targeted allele was crossed with transgenic mice expressing tissue specific Cre from the α-MHC promoter (α-MHC-MerCreMer; cardiomyocyte specific). To delete Bmal1 in Cre-positive tissues, tamoxifen (30 mg/kg) was administered by intraperitoneal injection for five consecutive days at ZT2 followed by a one-week washout period. | NA | Bmal1 fl/fl mice |
| Zhang 2023 | Mice | C57BL/6J | M | 8 weeks | 32.21 | 3-7 per group | Cardiomyocyte Bmal1 KO | Bmal1flox/flox/α-MHCCre+/- mice were used for constructing Bmal1 KO mice via intraperitoneal injection of tamoxifen (50 mg/kg) for five consecutive days. | 12 weeks | Bmal1flox/flox mice |
| Zhong 2024 | Mice | C57BL/6N | M | 15 weeks | 24.75 | 6 per group | Sleep disruption | Chamber which is a circular cage equipped with a metal sweeping bar. During the light cycle (ZT0-12), the sweep bar swept along the bedding to induce brief arousals every 2 min, the sweep bar automatically shut off during the dark cycle (ZT12-0). | 5 weeks | No intervention |
| Zlobina 2021 | Rat | outbred white | M | 8-10 weeks | 225 ± 25 | 12 per group | Light exposure | 18:6 LD: 18 h of continuous light with illuminance of 500 lx and a 6-h-long dark period. | 21 days | Normal light (12:12 LD) |

Abbreviations: LD: light-dark; DD: constant darkness, KO: knockout; Clock: ;Bmal1: ; Clockmut: Clock mutant; M: male; F: female; NS: not specified; NA: not applicable

Supplementary Table 9: Summary of key findings

| **Study** | **Biomarkers** | **Key findings** |
| --- | --- | --- |
| **Cardiac Markers (n=14)** | | |
| **Impaired Excitability (n=1)** | | |
| Schroder 2015 | Cardiac potassium (K+) channel transcripts (-); Corrected QT interval (QTc) (+) | Only *Kcnd2* and *Kcnh2* transcripts exhibit a robust circadian oscillation in control hearts. The circadian pattern of the *Kcnh2* transcript, but not the *Kcnd2* transcript, was lost in Brain and muscle ARNT-like 1 knockout *(Bmal1* KO) hearts. However, the average 24-hour expression levels of several other cardiac K+ channel transcripts (which did not follow a circadian pattern) were lower in Bmal1 KO hearts. These data suggested that the cardiomyocyte molecular clock signaling might indirectly contribute to the expression of non-circadian K+ channel genes. This was accompanied by a prolongation in the heart rate corrected QT (QTc) interval during the light (resting)-phase of KO mice. |
| **Impaired Cardiac Contractility and Relaxation (n=12)** | | |
| Che 2024 | Ejection fraction (EF) (0) | No changes in EF were observed in animals following a 3-month light phase intervention where the light was advanced by 8h every 4 days. |
| Durgan 2011 | Sarcoendoplasmic reticulum calcium adenosine triphosphatase (SERCA2) protein (+)(-) | Protein expression of SERCA2 was increased in a simulated shift work model, where mice were introduced to a bi-weekly 12h phase shift in the light/dark cycle for a total of 16 weeks. However, expression of SERCA2 was significantly reduced in *Clockmut* mice. |
| Jing 2020 | Left ventricular internal diameter (LVID) (+); Fractional shortening (FS), EF, +dP/dT (-) | Constant light exposure for 4 weeks resulted in increased LVID and decreased FS, EF, +dP/dT, implying the myocardial contraction was weakened after constant light exposure. |
| Lefta 2012 | FS (-) | Bmal1⁻/⁻ mice had a significant decline in FS, indicating systolic dysfunction. At 36 weeks, FS was reduced by 24.3% compared to wild-type mice (24.6±1.16% vs. 32.5±1.06%, P<0.01). |
| Li 2020 | EF, FS (-); LVID (+) | *Bmal1* KO mice showed impaired cardiac contractility with a decreased fraction of shortening and ejection fraction, and increased LVID. |
| Song 2022 | Contractility (-) | Cardiomyocyte-specific *Nr1d1/Nr1d2* KO mice showed normal contractile function at 2.5 months of age. However, after 4.5 and 6 months, the KO mice displayed impaired contractile function with an enlarged LVID, and decreased EF and FS. |
| Sutovska 2021 | SERCA2 protein (-) | Exposure to artificial light at night (12 h light (150 lx) – 12 h dim light (1−2 lx)) for 5 weeks led to decreased expression of SERCA2 protein in exposed animals, (control: 0.53 ± 0.07; light at night: 0.46 ± 0.10, P = 0.067). |
| Tsai 2010 | EF, FS (+); LVID (0) | EF and FS were significantly higher in hearts of cardiomyocyte-specific Clock mutant mice compared with wild-type (WT) mice while no differences were observed in left ventricular internal diameters at diastole (LVIDd), systole (LVIDs). |
| Wang 2023 | E/A ratio (-) | E/A was decreased in a mice model with continuously rolling light cycles where the light cycle was delayed backwards by 4 h every 3 days for 8 weeks, suggesting that chronic rhythm disturbances could lead to impairment in diastolic heart function. |
| Yusifova 2023 | EF, FS, Fractional area change (FAC) (-) | Cardiomyocyte-specific *Bmal1* KO mice developed impaired systolic function as evidenced by lower EF, FS, and FAC compared to WT. |
| Zhang 2023 | E/A ratio (-); Isovolumic relaxation time (IVRT), E/e' ratio, Left ventricular end-systolic diameter (LVESD), Left ventricular end-diastolic diameter (LVEDD) (+); EF, FS (0) | *Bmal1* KO in cardiomyocytes led to left ventricular dilation as shown by increased LVESD and LVEDD. Diastolic dysfunction was indicated by a decreased E/A ratio and elevated IVRT and E/e′ ratio. No significant changes were observed in EF or FS. |
| Zhong 2024 | EF, FS (-); End-diastolic volume (EDV) (0); End-systolic volume (ESV) (+) | ESV was significantly higher in mice subjected to sleep deprivation. The reduction of EF%, FS%, revealed impaired left ventricular contractile function. Specifically, EF% and FS% decreased by 15.8% and 19.3%, respectively, while ESV increased by 97.6% following 5 weeks of interventions compared with the control group. EF(%): intervention: 56.08 ± 8.74, control: 66.57 ± 2.72, p=0.019, (n=6); FS (%): intervention: 29.21 ± 5.93, control: 36.19 ± 2.06, p=0.033, (n=6); ESV (ul): intervention: 34.27 ± 14.15, control:17.34 ± 2.12, p=0.049 (n=6). |
| **Cardiomyocyte Injury and Death (n=10)** | | |
| Che 2024 | Interventricular septum thickness at diastole (IVSd), Left ventricular posterior wall thickness at diastole (LVPWd), Left ventricular mass to body weight ratio (LV/BW) (+) | The left ventricular weight normalized by body weight showed a significant increase in the jetlag group after 3 months. There was also significantly increased IVSd, LVPWd, and corrected LV/BW in the systolic function of jetlag mice with preserved ejection fraction. |
| Durgan 2011 | Cardiomyocyte size, Atrial natriuretic factor (*Nppa*), Myocyte-enriched calcineurin-interacting protein (*Rcan1*), Myosin heavy chain beta (*Myh7*) and Myosin heavy chain alpha (*Myh6*), Biventricular weight-to-body weight ratio (+) | Cardiomyocyte size and expression of *Nppa*, *Rcan1*, *Myh6* and *Myh7* were increased in *Clockmut* mice. The biventricular weight-to-body weight ratio was significantly elevated in both *Clockmut* and *Bmal1* KO mice. Septal wall thickness was increased in *Clockmut* mice. |
| Jing 2020 | LVPWd (0) | Rats exposed to constant light did not show any significant effect on LVPWd |
| Lefta 2012 | Interventricular septum (IVS), Left ventricular posterior wall (LVPW) thickness (-); Cardiomyocyte size, LV/BW (+) | Global *Bmal1* KO mice showed decreased IVS and LVPW thickness and increased cardiomyocyte size and LV/BW. |
| Li 2020 | IVS and LVPW thickness (-); Cardiomyocyte size (+) | At 32 weeks, *Bmal1* KO mice showed a significant thinning of both the interventricular septum (IVSs/IVSd) and posterior wall (LVPWs/LVPWd), indicating dilation and wall thinning. Myocyte cross-sectional area was increased, reflecting ventricular dilation. |
| Sato 2023 | Dystrophin (-) | Dystrophin protein levels were reduced in hearts of *Myh6-Cre*;*Bmal1flx/flx* mice compared to control *Bmal1flx/flx* mice. |
| Song 2022 | LV diameter, Atrial natriuretic peptide (ANP), B-type natriuretic peptide (BNP) (+) | Cardiomyocyte-specific *Nr1d1/Nr1d2*  mice showed normal LVPW thickness but developed progressive contractile dysfunction from 4.5 to 6 months of age, accompanied by an enlarged LV chamber and elevated ANP and BNP levels. These changes reflected pathological cardiac remodeling and were consistent across sexes and inducible deletion models. |
| Tsai 2010 | Interventricular septal thickness at systole (IVSs) (0); LVPWd (+) | Left ventricular posterior wall thickness in diastole was significantly higher in cardiomyocyte-specific *Clockmut* (CCM) mice compared to wild-type, while no differences were observed in IVSs. |
| Yusifova 2023 | LV/BW, Cardiomyocyte size, ANP, Fibrosis (+) | Cardiac-specific deletion of *Bmal1* resulted in increased LV mass/BW, cardiomyocyte size, cardiac fibrosis and ANP expression in *Bmal1* KO mice compared to WT. |
| Zhang 2023 | LVPWd, LV/BW, Cleaved poly(ADP-ribose) polymerase (cPARP), Cleaved caspase 9 (cCASP9), Cleaved caspase 3 (cCASP3), Cytochrome C-Mitochondrial (Cyto-C (Mito)), Cytochrome C-Cytosolic (Cyto-C (Cyto)), Second mitochondria-derived activator of caspase-Mitochondrial (Smac (Mito)), Second mitochondria-derived activator of caspase-Cytosolic (Smac (Cyto)), Apoptosis Positive Cells (TUNEL) (+) | Cardiac-specific *Bmal1* KO mice showed increased LVPW thicknesses (systolic and diastolic), LV/BW. Apoptosis markers (cPARP, cleaved caspase-3/9), cytochrome C release, and Terminal deoxynucleotidyl transferase dUTP nick end labeling (TUNEL)-positive cells were elevated. |
| Zhong 2024 | LV/BW, Left ventricular posterior wall thickness at diastole (LVPWd), IVSd (0) | LV/BW, LVPWd, and IVSd did not show statistically significant changes in mice subjected to sleep fragmentation. |
| **Vascular Markers (n=18)** | | |
| **Impacts on Endothelial and Vascular Function (n=8)** | | |
| Anea 2009 | Collagen deposition, Vasodilation (+); Acetylcholine-induced relaxation (-); Endothelium-independent vasodilation (0) | Studies in aged *Bmal1* KO revealed severe abnormalities in vascular remodeling accompanied by a substantial increase in collagen deposition in the medial layer. *Clockmut* mice under constant darkness (DD) conditions (not under light-dark (LD) conditions) exhibited pathological vascular responses manifested as increased wall thickness relative to WT mice following carotid artery ligation. *Clockmut* mice (DD) also had increased arterial injury relative to WT mice. Isolated aortic rings from *Bmal1* KO mice showed impaired endothelium-dependent vasorelaxant response to acetylcholine relative to WT mice. A similar response was observed in Clockmut DD mice. |
| Duan 2022 | Blood pressure (BP) (+); Heart rate (HR) (0) | Compared to rats within LD group, the mean arterial pressure (MAP, 96.9 ± 2.8 mmHg vs 123.0 ± 6.2 mmHg, P < 0.05) of rats within circadian disruption (CD) group were significantly increased. However, HR was not changed after the circadian disruption. |
| Durgan 2011 | BP (0) | BP was not affected in mice subjected to shift work simulation or Clockmut. |
| Hemmeryckx 2011 | Leukocyte rolling (-) | Lower numbers of rolling cells were found on *Bmal1* KO than WT mouse endothelium at all ages. |
| Kadomatsu 2013 | Angiopoietin-like protein 2 (*Angptl2*) rhythmicity abolished | Periodic *Angptl2* gene and protein expression were abolished in the aorta of Cry-deficient mice. These results indicate that components of a molecular clock are essential to regulate rhythmic Angptl2 expression in aorta. |
| Sutovska 2021 | Endothelin-1 (ET1), Angiotensin type 1 receptor (AT1R) (-) | Two weeks of artificial light at night (ALAN) did not affect ET1 protein expression but significantly (P = 0.028) decreased protein expression of AT1R in the left ventricle (control: 0.51 ± 0.27; ALAN: 0.34 ± 0.20). After 5 weeks of ALAN, decreased ET1 expression (P = 0.015) (control: 0.51 ± 0.27; ALAN: 0.28 ± 0.12) was observed. |
| Viswambharan 2007 | Endothelium-dependent relaxation, Mean arterial pressure (-) | Mice with *Per2* mutation exhibited impaired endothelium-dependent relaxations to acetylcholine in aortic rings. During transition from the inactive to active phase, this response was further increased in the WT mice but further decreased in the *Per2* mutants. Mean arterial pressure for the whole-day period (22 hours) in *Per2* mutant animals (105.9±1.9 mm Hg; n=10) was significantly lower than that in their WT counterparts (116.5±2.1 mm Hg; n=9; P=0.05). |
| Wang 2023 | Endothelium-dependent diastolic function, Coronary flow velocity reserve (CFVR) (-); Non-endothelium and endothelial dependent systolic and diastolic function (0) | Disruption of the circadian rhythm by delaying the light phase did not affect systolic and non-endothelium-dependent diastolic functions, but significantly reduced the endothelium-dependent diastolic function, showing that disruption of circadian rhythm led to diastolic heart function diminution. The intervention also led to significantly lower CFVR in exposed animals. Moreover, the E/A was decreased in the exposed group, suggesting that chronic rhythm disturbances could lead to impairment in diastolic heart function. |
| **Effects on Hemostasis (n=5)** | | |
| Anea 2009 | Thrombosis, Plasminogen activator inhibitor-1 (PAI1) (+); Platelet activation (0) | *Bmal1* KO mice (aged 25–30 weeks) exhibited significantly increased susceptibility to thrombosis in the ligated vessels compared to contralateral controls. PAI-1 protein expression was elevated in the endothelium of remodeled arteries. However, no differences were observed in platelet activation markers between KO and WT mice. |
| Cheng 2021 | Activated protein C (APC) (-); Fibronectin (0) | *Clock* knockdown mice had lower concentrations of APC in blood plasma than WT mice. |
| Hemmeryckx 2011 | Activated partial thromboplastin time (aPTT), Coagulation factor VII (FVII), Fibrinogen (+); Prothrombin time (PT), Tissue factor (TF), von Willebrand factor (VWF), Thrombomodulin (TM), Endothelial protein C receptor (EPCR) (-) | *Bmal1* gene deficiency was associated with a prolongation of the aPTT and a shortening of the PT by 1.6 seconds in 10-week-old mice and 0.8 seconds in 30-week KO mice, compared with their age-matched controls. At 10 weeks aPTT was increased and PT levels were decreased in KO mice. At 30 weeks, platelet number, aPTT, plasma FVII and Fibrinogen levels were increased and PT levels were decreased in KO mice compared to WT mice. Vascular TF, VWF, TM and EPCR were reduced in KO mice at 30 weeks. No effects were observed at 10 weeks. Bmal1 deficiency shortened arteriolar as well as venular occlusion times. |
| Shi 2022 | Tail-bleeding time (+); P-selectin, Fibrinogen-binding, JON/A-binding, Clot retraction (-); Platelet count, Platelet size, aPTT, PT, Platelet granules (α-granules, dense granules), CD41, GPVI surface expression (0) | *Nr1d1* KO mice exhibited significantly prolonged tail bleeding time and impaired platelet aggregation. There was no change in platelet counts, platelet size, aPTT, PT, CD41, or GPVI expression. However, platelet-specific KO mice had reduced P-selectin surface expression, decreased fibrinogen- and JON/A-binding, and significantly decreased clot retraction in platelet-rich plasma (PRP). These findings indicate that *Nr1d1* deletion impairs platelet activation and thrombus formation despite preserved coagulation times and platelet counts. |
| Zlobina 2021 | Maximum Size of Platelet Aggregates, Maximum Rate of Platelet Aggregate Formation, Time to Achieve Maximum Size, Maximum Degree of Aggregation (Day 10), Maximum Rate of Aggregation (Day 10), Time to Achieve Maximum Degree of Aggregation (+); Maximum Degree of Aggregation (Day 21), Maximum Rate of Aggregation (Day 21) (-); Time to Achieve Maximum Rate (0) | Maximum size of platelet aggregates, maximum rate of platelet aggregate formation, and time to achieve maximum size were increased at both day 10 and day 21 of prolonged photoperiod exposure. Time to achieve maximum rate showed no change at day 10 but was increased at day 21. The time to achieve maximum degree of aggregation increased significantly at both timepoints (22% at day 10, 58% at day 21). However, both maximum degree and rate of aggregation decreased by 15% and 10%, respectively, at day 21 compared to day 10, indicating a biphasic response under sustained circadian disruption. |
| **Dyslipidemia (n=11)** | | |
| Anea 2009 | Triglycerides (TG) (+); Total cholesterol (TC) (0) | TG were markedly elevated in *Bmal1* KO mice across all age and sex groups compared to wild-type controls. In young male mice, TG levels increased from 28.9 ± 8.1 mg/dL in WT to 126 ± 20 mg/dL in KO. Similar elevations were observed in old males (WT: 52.4 ± 6.8, KO: 79.1 ± 18.3), young females (WT: 43.0 ± 10.1, KO: 83.1 ± 14.9), and old females (WT: 49.3 ± 10.9, KO: 86.1 ± 35.7). Total cholesterol showed a mild increase or no effect across age groups, with small differences between WT and KO mice in each sex/age category. |
| Durgan 2006 | TG, Free fatty acids (FFA) (0) | No significant differences were observed in plasma FFA levels or cardiac TG content in ad libitum-fed CCM mice compared to wild-type controls, indicating that circadian *Clock* mutation alone did not alter lipid metabolism under these conditions. |
| Durgan 2011 | TG (+) | Triglyceride levels were higher in the stimulated shift work group than controls independent on age and genotype. |
| Escribano 2014 | TC (0) | Cholesterol levels in blood were not affected by photoperiod when compared with the control. However, light exposed animals had significantly lower cholesterol than dark exposed animals. |
| Ferrell 2015 | TC, TG (-) | The diurnal daytime elevation in serum cholesterol was statistically significantly lower in sleep disturbed mice. Similarly, serum triglyceride levels were statistically significantly suppressed by sleep disruption early in the day at Zeitgeber time (ZT) 2. |
| Gu 2024 | Low-density lipoprotein cholesterol (LDL-C) (+), TC, High-density lipoprotein cholesterol (HDL-C), TG, FFA (0) | Circadian disturbance led to an increase in LDL-C levels but did not significantly change TG, FFA, TC and HDL-C levels. |
| Mia 2020 | TG (0) | The net TG synthesis did not differ between the *Bmal1* KO and WT mice. |
| Oishi 2006 | TG (-); TC (0) | Reduced levels of serum TG were found in *Clock* mutant mice but not TC. |
| Pan 2013 | TG, TC, HDL-C, Lipid lesions (+) | *Clock*Δ19/Δ19 mice displayed elevated plasma triglycerides, total cholesterol, and HDL compared to WT controls under chow-fed conditions. Upon 2 months of atherogenic diet, *Clock*Δ19/Δ19 mice showed 2- to 3-fold increases in plasma cholesterol and triglycerides, primarily involving very-low-density lipoprotein/intermediate-density lipoprotein/low-density lipoprotein (VLDL/IDL/LDL) fractions, and developed significantly more lipid lesions in the aortic arch, root, and abdominal aorta. |
| Tsai 2010 | Diacylglycerol acyltransferase 2 (DGAT2), Hormone-sensitive lipase (HSL), s3-12 (-) | On a control diet, CCM hearts exhibit reduced dgat2, hsl, and s3-12 mRNA levels relative to WT hearts. In addition, high fat feeding increased dgat2, hsl mRNA in CCM, but not WT, hearts. High fat diet also led to enhanced TG levels in plasma and a greater accumulation of myocardial triglyceride levels in CCM, versus WT, mice. In control-fed animals no effects of genotype was observed. |
| Viswambharan 2007 | TC, TG (0) | No difference in plasma concentrations of total cholesterol and triglyceride was observed between WT and Per2 mutant mice. |
| **Cardiovascular Markers (n=14)** | | |
| **Mitochondrial Dysfunction (n=2)** | | |
| Escribano 2014 | Total glutathione (GLT), Reduced glutathione (GSH), Glutathione peroxidase (Gpx), Glutathione reductase (GRd), Glutathione transferase (GST), Catalase (CAT) (-) | Light exposure resulted in reduced levels of GLT, GSH, CAT, Gpx, GRd, and GST in the heart. |
| Mia 2020 | Mitochondrial complex proteins | No significant differences were observed in mitochondrial complex I, II, or III activity between cardiomyocyte-specific *Bmal1* knockout (CBK) and WT hearts. Complex II levels were slightly reduced, while complex IV levels were marginally higher in CBK mice. Complex IV activity was increased, though not statistically significant. |
| **Alterations in Autonomic Nervous System Activity (n=4)** | | |
| Duan 2022 | Plasma norepinephrine (NE) (+) | Plasma level of NE (118.3 ± 4.4 ng/ml vs 142.6 ± 3.4 ng/ml, P < 0.05) of rats within CD group were significantly increased compared to the control animals. |
| Hou 2025 | Impairment of urinary NE rhythmicity | Rhythmicity of urinary NE expression was abolished in inducible *Bmal1* KO mice under constant dark. |
| Jing 2020 | Plasma NE (+) | Constant light exposure increased the plasma NE levels compared to animals in 12:12 LD cycle. |
| Sutovska 2021 | Tyrosine hydroxylase (TH) (0) | TH was not affected by artificial light at night exposure after 2 (P = 0.461) or 5 weeks (P = 0.240). |
| **Oxidative Stress (n=7)** | | |
| Anea 2009 | Endothelial nitric oxide synthase (eNOS) (-) | The levels of phosphorylated eNOS were significantly attenuated in *Bmal1* KO mice relative to WT mice. |
| Anea 2013 | NADPH oxidase 4 (Nox4) (+); NADPH oxidase 1 (Nox1) (0) | Nox4 expression was significantly elevated (3.5-fold) in the aorta of *Bmal1* KO mice compared to WT controls, while Nox1 expression did not differ. These results suggest circadian disruption via Bmal1 deletion increases vascular oxidative stress through Nox4 upregulation in vivo. |
| Che 2024 | eNOS (-); Inducible nitric oxide synthase (iNOS), Neuronal nitric oxide synthase (nNOS) (0) | eNOS expression at the protein level decreased in the jetlag group after 1 month at light phase while iNOS and nNOS did not alter. |
| Escribano 2014 | Lipid peroxidation (LPO) (+) | LPO levels were increased in the heart of light exposed animals. |
| Hemmeryckx 2011 | eNOS, iNOS (0) | *Bmal1* KO did not affect eNOS and iNOS expression levels in the heart. |
| Periasamy 2015 | Serum antioxidant activity (-) | Serum antioxidant activity was significantly lower in all three sleep deprivation (SD) groups than in controls. Heart antioxidant levels were significantly higher only in SD1 group mice and significantly lower in SD3 mice. |
| Viswambharan 2007 | Cyclooxygenase-1 (COX1) (+) | No differences in eNOS but increased COX1 (not cyclooxygenase-2) protein levels in the aortas of WT and *Per2* mutant mice were observed. |
| **Inflammation (n=4)** | | |
| Hemmeryckx 2011 | White blood cell (WBC) count, Neutrophils, C-reactive protein (CRP) (+) | *Bmal1* KO mice had increased WBC counts and neutrophil numbers 2-fold at 10 weeks. CRP was somewhat higher in Bmal1-deficient mice versus WT mice at 10 weeks and 30 weeks. |
| Periasamy 2015 | Serum interleukin-1 beta (IL-1β), Serum interleukin-6 (IL-6), Cardiac IL-1β, Cardiac tumor necrosis factor alpha (TNF-α) (+) | Acute and prolonged sleep deprivation induced inflammatory changes in serum and cardiac tissue. Serum IL-1β levels were significantly elevated after 24 h of sleep deprivation (SD1) but decreased below control levels following 72 h (SD3). IL-6 was undetectable in controls but significantly increased in SD1 and reached peak levels in SD3 mice. Cardiac TNF-α and IL-1β were significantly elevated in both SD1 and SD2 mice compared to controls, indicating tissue-level inflammation in early stages of sleep loss. |
| Song 2022 | C-C motif chemokine ligand 2 (*Ccl2*), C-C motif chemokine receptor 2 (*Ccr2*) and C-C motif chemokine receptor 5 (*Ccr5*) (+) | Inflammatory genes in cardiac-specific *Nr1d1/Nr1d2* double KO hearts were not upregulated at 2 months of age but at 4.5 months of age *Ccl2*, *Ccr2* and *Ccr5* expression was increased in hearts of male KO mice compared with WT mice. |
| Wang 2023 | Cell-free DNA (cfDNA), Myeloperoxidase (MPO), Neutrophil extracellular trap (NET) (+) | Sleep disruption significantly increased NET formation in vivo. Plasma levels of NETs markers—cfDNA and MPO—were significantly elevated within 24 h in the sleep disruption model group compared to controls. Ex vivo neutrophils from the model group also showed a higher rate of NETs formation based on PicoGreen staining, confirming enhanced NETs activity. |

Supplementary Table 10: Assessment of GRADE criteria

| **Biomarkers** | **Bias Score** | **Inconsistancy** | **Imprecision** | **Publication bias** | **Certainty of evidence** | **Comment** |
| --- | --- | --- | --- | --- | --- | --- |
| Cardiomyocyte size | L(0) | L(0) | L(0) | NA | ⊕⊕⊕◯ | Moderate – Lack of quantitative analysis (-1) |
| EF | L(0) | L(0) | M(-1) | M(-1) | ⊕⊕◯◯ | Low – CI crossed null; very high heterogeneity (I²=86.1%); sensitivity analysis showed effect but I²>75% remained |
| FS | L(0) | L(0) | M(-1) | M(-1) | ⊕⊕◯◯ | Low – CI crossed null; very high heterogeneity (I²=84.5%); sensitivity analysis showed effect but I²>75% remained |
| IVS | L(0) | M(-1) | M(-1) | NA | ⊕⊕◯◯ | Low – High heterogeneity (I²=83.4%); wide CI crossing null; only 4 studies with inconsistent directions |
| LV/BW | L(0) | L(0) | L(0) | L(0) | ⊕⊕⊕⊕ | High – Low heterogeneity (I²=0%); precise estimate; consistent across genetic models |
| LVID | L(0) | M(-1) | M(-1) | M(-1) | ⊕◯◯◯ | Very low – high heterogeneity (I²=91.2%); wide CI crossing null; variable effects across models |
| LVPWd | L(0) | M(-1) | M(-1) | M(-1) | ⊕◯◯◯ | Very low – High heterogeneity (I²=81.6%); wide CI crossing null; opposite effects in global vs tissue-specific models |
| TC | L(0) | M(-1) | M(-1) | NA | ⊕⊕◯◯ | Low – High heterogeneity (I²=88.8%); wide CI crossing null; only 4 studies |
| TG | L(0) | M(-1) | L(0) | M(-1) | ⊕⊕◯◯ | Low – Very high heterogeneity (I²=90.9%); only 6/10 studies had extractable data; excluded studies showed opposite effects |
| Vasorelaxation | L(0) | M(-1) | L(0) | M(-1) | ⊕⊕◯◯ | Low – High heterogeneity (I²=86.4%); only 3 studies, all genetic models; no environmental validation |

The evidence was downgraded by (-1) if overall bias of included studies had moderate risk of bias. The evidence was downgraded by (-1) for inconsistency or heterogeneity, assessed based on differing effect estimates or I^2^ statistics, if I^2^ >75% and if no plausible explanation was identified by subgroup or sensitivity analyses. The evidence was downgraded by (-1 or -2) for imprecision if estimates were based on few animals (n≤50) or the data had wide confidence intervals. Publication bias was assessed based on visual inspection of funnel plots and downgraded by (-1) if publication bias or outliers outside the 95% CI were present. For indicators with <4 studies or lacking quantitative synthesis, publication bias was not assessed

## **References**

1. Page MJ, Moher D, Bossuyt PM, Boutron I, Hoffmann TC, Mulrow CD, Shamseer L, Tetzlaff JM, Akl EA, Brennan SE *et al*: **PRISMA 2020 explanation and elaboration: updated guidance and exemplars for reporting systematic reviews**. *BMJ* 2021, **372**:n160.

2. Lind L, Araujo JA, Barchowsky A, Belcher S, Berridge BR, Chiamvimonvat N, Chiu WA, Cogliano VJ, Elmore S, Farraj AK *et al*: **Key Characteristics of Cardiovascular Toxicants**. *Environmental Health Perspectives* 2021, **129**(9):095001.
